# Neurological and psychiatric risk trajectories after SARS-CoV-2 infection: an analysis of 2-year retrospective cohort studies including 1 284 437 patients

**DOI:** 10.1016/S2215-0366(22)00260-7

**Published:** 2022-10

**Authors:** Maxime Taquet, Rebecca Sillett, Lena Zhu, Jacob Mendel, Isabella Camplisson, Quentin Dercon, Paul J Harrison

**Affiliations:** a Department of Psychiatry, University of Oxford, Oxford, UK; b Medical Sciences Division, University of Oxford, Oxford, UK; c Oxford Health NHS Foundation Trust, Oxford, UK; d MRC Cognition and Brain Sciences Unit, University of Cambridge, UK

## Abstract

**Background:**

COVID-19 is associated with increased risks of neurological and psychiatric sequelae in the weeks and months thereafter. How long these risks remain, whether they affect children and adults similarly, and whether SARS-CoV-2 variants differ in their risk profiles remains unclear.

**Methods:**

In this analysis of 2-year retrospective cohort studies, we extracted data from the TriNetX electronic health records network, an international network of de-identified data from health-care records of approximately 89 million patients collected from hospital, primary care, and specialist providers (mostly from the USA, but also from Australia, the UK, Spain, Bulgaria, India, Malaysia, and Taiwan). A cohort of patients of any age with COVID-19 diagnosed between Jan 20, 2020, and April 13, 2022, was identified and propensity-score matched (1:1) to a contemporaneous cohort of patients with any other respiratory infection. Matching was done on the basis of demographic factors, risk factors for COVID-19 and severe COVID-19 illness, and vaccination status. Analyses were stratified by age group (age <18 years [children], 18–64 years [adults], and ≥65 years [older adults]) and date of diagnosis. We assessed the risks of 14 neurological and psychiatric diagnoses after SARS-CoV-2 infection and compared these risks with the matched comparator cohort. The 2-year risk trajectories were represented by time-varying hazard ratios (HRs) and summarised using the 6-month constant HRs (representing the risks in the earlier phase of follow-up, which have not yet been well characterised in children), the risk horizon for each outcome (ie, the time at which the HR returns to 1), and the time to equal incidence in the two cohorts. We also estimated how many people died after a neurological or psychiatric diagnosis during follow-up in each age group. Finally, we compared matched cohorts of patients diagnosed with COVID-19 directly before and after the emergence of the alpha (B.1.1.7), delta (B.1.617.2), and omicron (B.1.1.529) variants.

**Findings:**

We identified 1 487 712 patients with a recorded diagnosis of COVID-19 during the study period, of whom 1 284 437 (185 748 children, 856 588 adults, and 242 101 older adults; overall mean age 42·5 years [SD 21·9]; 741 806 [57·8%] were female and 542 192 [42·2%] were male) were adequately matched with an equal number of patients with another respiratory infection. The risk trajectories of outcomes after SARS-CoV-2 infection in the whole cohort differed substantially. While most outcomes had HRs significantly greater than 1 after 6 months (with the exception of encephalitis; Guillain-Barré syndrome; nerve, nerve root, and plexus disorder; and parkinsonism), their risk horizons and time to equal incidence varied greatly. Risks of the common psychiatric disorders returned to baseline after 1–2 months (mood disorders at 43 days, anxiety disorders at 58 days) and subsequently reached an equal overall incidence to the matched comparison group (mood disorders at 457 days, anxiety disorders at 417 days). By contrast, risks of cognitive deficit (known as brain fog), dementia, psychotic disorders, and epilepsy or seizures were still increased at the end of the 2-year follow-up period. Post-COVID-19 risk trajectories differed in children compared with adults: in the 6 months after SARS-CoV-2 infection, children were not at an increased risk of mood (HR 1·02 [95% CI 0·94–1·10) or anxiety (1·00 [0·94–1·06]) disorders, but did have an increased risk of cognitive deficit, insomnia, intracranial haemorrhage, ischaemic stroke, nerve, nerve root, and plexus disorders, psychotic disorders, and epilepsy or seizures (HRs ranging from 1·20 [1·09–1·33] to 2·16 [1·46–3·19]). Unlike adults, cognitive deficit in children had a finite risk horizon (75 days) and a finite time to equal incidence (491 days). A sizeable proportion of older adults who received a neurological or psychiatric diagnosis, in either cohort, subsequently died, especially those diagnosed with dementia or epilepsy or seizures. Risk profiles were similar just before versus just after the emergence of the alpha variant (n=47 675 in each cohort). Just after (*vs* just before) the emergence of the delta variant (n=44 835 in each cohort), increased risks of ischaemic stroke, epilepsy or seizures, cognitive deficit, insomnia, and anxiety disorders were observed, compounded by an increased death rate. With omicron (n=39 845 in each cohort), there was a lower death rate than just before emergence of the variant, but the risks of neurological and psychiatric outcomes remained similar.

**Interpretation:**

This analysis of 2-year retrospective cohort studies of individuals diagnosed with COVID-19 showed that the increased incidence of mood and anxiety disorders was transient, with no overall excess of these diagnoses compared with other respiratory infections. In contrast, the increased risk of psychotic disorder, cognitive deficit, dementia, and epilepsy or seizures persisted throughout. The differing trajectories suggest a different pathogenesis for these outcomes. Children have a more benign overall profile of psychiatric risk than do adults and older adults, but their sustained higher risk of some diagnoses is of concern. The fact that neurological and psychiatric outcomes were similar during the delta and omicron waves indicates that the burden on the health-care system might continue even with variants that are less severe in other respects. Our findings are relevant to understanding individual-level and population-level risks of neurological and psychiatric disorders after SARS-CoV-2 infection and can help inform our responses to them.

**Funding:**

National Institute for Health and Care Research Oxford Health Biomedical Research Centre, The Wolfson Foundation, and MQ Mental Health Research.

## Introduction

Since the early stages of the pandemic, COVID-19 has been known to be associated with an increased risk of many neurological and psychiatric sequelae.^1^, ^2^, ^3^, ^4^, ^5^ However, more than 2 years after the first case was diagnosed, three important questions remain unanswered.

First, we do not know if or when the risks of different post-COVID-19 outcomes return to baseline. This information is important to patients (who want to know when they can stop worrying about potential complications of their SARS-CoV-2 infection), clinicians (who need to know whether a clinical presentation is plausibly attributable to a post-COVID condition), and health policy makers (who must plan appropriate service provision). Previous studies on the neurological and psychiatric sequelae of COVID-19 did not investigate this question.^3^, ^4^, ^5^, ^6^ One study estimated the prevalence of self-reported anxiety, depressive, and sleep symptoms at 2, 2–6, and 6–16 months after COVID-19 diagnosis and found that the prevalence of depressive symptoms, but not sleep symptoms, decreased over time.^7^ Although informative, the self-reported nature of these findings and the restricted range of outcomes measured limit their usefulness for clinical practice and for informing public health policies.

**Research in context**

**Evidence before this study**

We searched PubMed (Medline) on March 21, 2022, for publications since database inception in English using the terms “(neuropsychiatr*[Title/Abstract] OR neurologic*[Title/Abstract] OR psychiatric[Title/Abstract] OR depress*[Title/Abstract] OR anxiety*[Title/Abstract] OR cognit*[Title/Abstract] OR brain[Title/Abstract]) AND (variant*[Title/Abstract] OR omicron[Title/Abstract] OR delta[Title/Abstract] OR evolution[Title/Abstract]) AND (COVID[Title/Abstract] OR COVID-19[Title/Abstract] OR SARS*[Title/Abstract])”. We found cohort studies and systematic reviews reporting neuropsychiatric sequelae persisting up to 10 months after COVID-19. We found one large, 6-month electronic health records study of neuropsychiatric disorders after a COVID-19 diagnosis. This study reported increased incidence and relative risk of cognitive symptoms and anxiety or depression 6 months after COVID-19, compared with influenza. We are not aware of any large-scale data regarding long-term COVID-19 sequelae beyond 12 months, or the evolution of incidence or relative risk of neuropsychiatric diagnoses in patients recovered from COVID-19 throughout the pandemic, stratified by COVID-19 variant or vaccination status.

**Added value of this study**

To our knowledge, this is the first study with a comparator cohort that assesses the risks of a range of neurological and psychiatric outcomes of COVID-19 up to 2 years after the index SARS-CoV-2 infection. We found that the risks of post-COVID neurological and psychiatric outcomes follow different trajectories: the risk of cognitive deficit, dementia, psychotic disorder, and epilepsy or seizures remain elevated 2 years after SARS-CoV-2 infection, while the risks of other diagnoses (notably, mood and anxiety disorders) subside after 1–2 months and show no overall excess over the whole 2-year follow-up. We also found that risk trajectories differ somewhat in children: they are not at an increased risk of mood or anxiety disorders (even over the first 6 months) and their risk of cognitive deficit is transient, but they share adults’ risk of several other diagnoses and are notably at risk of epilepsy or seizures. Finally, we found that the risks of neurological and psychiatric outcomes remain similar after the emergence of the omicron (B.1.1.529) variant as with the delta (B.1.617.2) variant, but are offset by a significantly lower death rate.

**Implications of all the available evidence**

The persisting increased risk of post-COVID-19 cognitive deficit, dementia, psychotic disorders, and epilepsy or seizures 2 years after the index infection calls for enhanced service provision to diagnose and manage these sequelae, and research to understand the mechanisms. The differing profile of post-COVID-19 neurological and psychiatric diagnoses in children informs the risk–benefit association of policies aimed at preventing COVID-19 in paediatric populations and suggests that underlying mechanisms might in part be different from those in adults. The observation of comparable neurological and psychiatric risks just after (compared with just before) emergence of the omicron variant suggests an ongoing neuropsychiatric burden of COVID-19 even with variants that lead to otherwise less severe disease.

Second, the risk profile in different age strata, especially in children, has not been well characterised.^8^ Neurological manifestations of COVID-19 in paediatric populations (aged <18 years) have been reported,^9^ but only one controlled study has investigated the risk of neurological and psychiatric outcomes after SARS-CoV-2 infection.^10^ This study was limited to a 3-month follow-up period and measured neurological and psychiatric outcomes as two broad categories, without reporting the risk of individual diagnoses.

Third, whether or not risk profiles have changed with the emergence of different variants is unknown; in particular, whether or not the omicron (B.1.1.529) variant, which has a lower mortality rate and better acute outcomes than the alpha (B.1.1.7) and delta (B.1.617.2) variants^11^, ^12^, ^13^ and a different symptom profile,^14^ also leads to fewer neurological and psychiatric sequelae.

We used electronic health records to investigate these three questions. We assessed the 2-year risk trajectories of 14 neurological and psychiatric diagnoses in three age groups (children younger than 18 years, adults aged 18–64 years, and older adults aged ≥65 years), and if and when these risks returned to baseline. Then we compared these risks between patients diagnosed just after versus just before the emergence of the alpha, delta, and omicron variants.

## Methods

### Study design, population, and data collection

In this analysis of retrospective cohort studies, we used two electronic health records networks. The primary source was the TriNetX Analytics Network a federated network recording anonymised data from electronic health records of approximately 89 million patients in 62 health-care organisations located mainly in the USA but also in Australia, the UK, Spain, Bulgaria, India, Malaysia, and Taiwan. The participating health-care organisations in the networks are hospitals, primary care, and specialist providers who contribute data from uninsured and insured patients. From this data source we compiled our primary cohorts of patients with COVID-19 and any other respiratory infection. The secondary network was TriNetX's US Collaborative Network (comprising approximately 78 million patients), which is restricted to the USA. We used this network when comparing outcomes just after versus just before the emergence of new SARS-CoV-2 variants, because the time of emergence of variants varied across countries. Both networks contain the same range of data, including demographics, diagnoses represented by ICD-10 codes, medications, and procedures. Our study design was similar to that described in previous publications using the TriNetX platform,^4^, ^15^ although with several modifications reflecting the longer duration of follow up and the different questions being addressed (appendix pp 2–3). Additional details about TriNetX are in the appendix (pp 1–2).

Using the TriNetX user interface, we created cohorts based on our inclusion and exclusion criteria. The primary cohort was defined as all patients who had a confirmed diagnosis of COVID-19 (ICD-10 code U07.1). We constructed a contemporaneous propensity-score matched comparator cohort of patients diagnosed with any other upper or lower respiratory infection (hereafter referred to as other respiratory infection) who did not have a COVID-19 diagnosis or a positive test for SARS-CoV-2 before their infection or during follow-up. The dates of diagnosis of COVID-19 and other respiratory infection are referred to as index events. The cohorts included patients of any age who had an index event on or after Jan 20, 2020 (the date of the first recorded COVID-19 case in the USA), up to the end of follow-up (April 13, 2022).

Using TriNetX's US Collaborative Network, we created primary alpha, delta, and omicron cohorts comprising patients who had a first diagnosis of COVID-19 within the time windows corresponding to the dominance of a particular SARS-CoV-2 variant, and the comparator pre-alpha, pre-delta, and pre-omicron cohorts comprising those with a first diagnosis of COVID-19 in the period just before the emergence of that variant. These time windows were defined to be minimally separated in time (to minimise the effect of contextual factors and seasonality in the USA) while retaining sufficient statistical power to detect group differences (appendix pp 8–9). As a result, the alpha cohort comprised patients diagnosed between March 22 and April 24, 2021 (with the pre-alpha cohort diagnosed between Feb 3 and March 8, 2021); the delta cohort was diagnosed between June 14 and Aug 5, 2021 (with the pre-delta cohort diagnosed between April 16 and May 31, 2021); and the omicron cohort was diagnosed between Dec 24 and Dec 31, 2021 (with the pre-omicron cohort diagnosed between Nov 25 and Dec 12, 2021). Two caveats might affect interpretation of results of the analysis of variants: variant-associated changes in service provision possibly affecting rates of neuropsychiatric diagnoses, and milder variants leading to only the more severely ill patients seeking care and subsequently receiving a diagnosis. However, we found no evidence of any substantial effect of these caveats (appendix pp 9–10). Rates of neuropsychiatric diagnoses remained largely unchanged in the general population (within TriNetX US Collaborative Network) in the follow-up time windows, and rates of COVID-19 diagnoses closely tracked the incidence of cases reported by the US Centers for Disease Control and Prevention.

Data de-identification is formally attested as per section §164.514(b)(1) of the Health Insurance Portability and Accountability Act Privacy Rule. Because we used anonymised and routinely collected data, no participant consent was required. Additional details on cohorts are provided in the appendix (pp 3–5). We followed the Reporting of studies Conducted using Observational Routinely-collected health Data (known as RECORD) guidelines (appendix pp 48–51).

### Propensity-score matching

The COVID-19 and other respiratory infection cohorts were stratified by age group (age <18, 18–64, and ≥65 years) and by date of the index events in 2-monthly periods. Within each stratum, cohorts were propensity-score matched (1:1) for 82 covariates: sociodemographic factors and comorbidities representing risk for COVID-19, for more severe COVID-19 illness,^16^ or for COVID-19 sequelae,^4^, ^15^ COVID-19 vaccination status, and previous or concurrent use of medications known to be associated with COVID-19 incidence or outcomes^17^, ^18^, ^19^ (ie, any antipsychotics [and clozapine specifically], any antidepressant [and fluvoxamine specifically], and lithium). Propensity-score matching with the same covariates was separately applied to the pairs of additional US COVID-19 variant cohorts (eg matching the omicron to the pre-omicron cohort). More details, including individual ICD-10 and medication codes, are in the appendix (pp 5–6).

We used a greedy nearest-neighbour matching approach with a caliper distance of 0·1 pooled SDs of the logit of the propensity score. Characteristics with a standardised mean difference (SMD) between cohorts lower than 0·1 were considered to be adequately matched.^20^

### Outcomes

We investigated neurological and psychiatric sequelae of COVID-19 in terms of 14 outcomes occurring up to 2 years after the index event or the last day of follow-up (April 13, 2022), whichever came first: anxiety disorder; mood disorder; psychotic disorder; insomnia; cognitive deficit (a composite of several ICD-10 F, G, and R codes to capture so-called brain fog; appendix p 7); dementia; epilepsy or seizures; encephalitis; intracranial haemorrhage; ischaemic stroke; parkinsonism; Guillain-Barré syndrome; nerve, nerve root, and plexus disorders; and myoneural junction (neuromuscular) and muscle disease. We also measured all-cause mortality, using the mortality data recorded in electronic health records or imported into TriNetX. When the number of deaths after a sequela was lower than 120 within an age group, the data were considered to be unreliable and therefore not reported (appendix p 8).

As in our previous study,^4^ for outcomes that are chronic illnesses (ie, dementia, myoneural junction or muscle disease, and parkinsonism), we excluded patients who had the diagnosis before the index event in the analysis of that outcome. For outcomes that often recur or relapse (ie, anxiety disorder, mood disorder, psychotic disorder, cognitive deficit, epilepsy or seizures, insomnia, intracranial haemorrhage, and ischaemic stroke), we took the same approach and focused on the incidence of first diagnosis; however, for completeness, we also report the incidence of any diagnosis (ie, including patients who had a diagnosis at some point before the index event). For outcomes that do not usually recur after they have resolved (ie, Guillain-Barré syndrome, encephalitis, and nerve, nerve root, or plexus disorders), we estimated the incidence of any diagnosis. To assess the overall risk of neurological and psychiatric outcomes after COVID-19, we estimated the incidence of a first diagnosis of any of the 14 outcomes. Finally, to account for death as a competing risk and to estimate the mortality in those diagnosed with a post-COVID-19 neurological or psychiatric disorder, we assessed the composite risks of death with each diagnosis (as well as death on its own) and the composite of any first diagnosis or death. More details on outcome definitions are in the appendix (pp 7–8).

### Statistical analysis

We used the Kaplan-Meier approach to estimate the incidence of each outcome (first instance, any instance, and composite with death, as appropriate). We made comparisons between the matched COVID-19 and other respiratory infection cohorts using the log-rank test. The 2-year risk trajectories were represented by time-varying hazard ratios (HRs) calculated using natural cubic splines fitted to the log-cumulative hazard.^21^ We also summarised these risk trajectories with three statistics for each outcome. The first statistic is the constant HR over the first 6 months calculated using the Cox proportional hazard model. This represents the risks in the earlier phase of follow-up (which have not yet been well characterised in children). The second statistic is a risk horizon defined as the time at which the time-varying HR reached 1 (ie, the point at which the risk of being diagnosed with the outcome became the same in the COVID-19 and other respiratory infection cohorts). The third statistic is the time to equal incidence defined as the time when the cumulative incidence was equal between the two matched cohorts (appendix p 9). We tested differences in cumulative incidence between the matched COVID-19 and other respiratory infection cohorts at the end of the follow-up period using a non-parametric bootstrap technique with 1000 replicates. We determined the proportion of risk associated with people who eventually died after a neurological or psychiatric diagnosis using simple algebra applied to cumulative incidences of individual outcomes and the composite of death or individual outcomes. We assessed the severity of infection in the matched cohorts in terms of the need for hospitalisation, intensive care admission, and mechanical ventilation within 14 days of the index event.

For the analysis of the risks of outcomes by SARS-CoV-2 variants, we calculated constant HRs over the whole follow-up (6-months for alpha and delta, 140 days for omicron) using the Cox proportional hazard ratio. As in the other analyses, both the individual outcomes and their composite with death are reported.

Additional details of the statistical analyses are in the appendix (pp 8–9). We did all analyses using R (version 3.6.3), except for propensity-score matching, which was done in TriNetX. We considered two-sided p values of less than 0·05 to be significant. We did not correct for multiple testing in the primary analyses (appendix p 9) but do also report corrected p values.

### Role of the funding source

The funder of the study had no role in study design, data collection, data analysis, data interpretation, or writing of the manuscript.

## Results

We extracted data for 1 487 712 patients with a recorded diagnosis of COVID-19 between Jan 20, 2020, and April 13, 2022, from the TriNetX Analytics database, of whom 1 284 437 were adequately matched (ie, standardised mean difference of <0·1 for each covariate) to an equal number of patients with another respiratory infection. Both matched cohorts comprised 185 748 children (aged <18 years), 856 588 adults (aged 18–64 years), and 242 101 older adults (aged ≥65 years). In the matched COVID-19 cohort, mean follow-up was 213 days (SD 204; median follow-up 146 days [IQR 20–364]), and in the matched other respiratory infection cohort, the mean follow-up was 223 days (SD 203; median 153 days [IQR 41–399]; table 1; appendix pp 15–26). In the matched COVD-19 cohort, 741 806 (57·8%) patients were female and 542 192 (42·2%) were male; in the matched comparator cohort with another respiratory infection, 741 696 (57·7%) were female and 542 305 (42·2%) were male.

**Table 1:** Baseline characteristics for the whole COVID-19 cohort and the matched cohorts of COVID-19 patients and patients diagnosed with another respiratory infection

		**COVID-19 cohort (unmatched; n=1 487 712)**	**COVID-19 cohort (propensity-score matched; n=1 284 437)**	**Other respiratory infection cohort (propensity-score matched; n=1 284 437)**
Age, years		44·0 (21·6)	42·5 (21·9)	42·6 (22·1)
Sex				
	Female	822 711 (55·3%)	741 806 (57·8%)	741 696 (57·7%)
	Male	664 460 (44·7%)	542 192 (42·2%)	542 305 (42·2%)
Race				
	White	832 557 (56·0%)	745 846 (58·1%)	745 452 (58·0%)
	Black or African American	250 764 (16·9%)	203 616 (15·9%)	203 086 (15·8%)
	Asian	36 464 (2·5%)	29 864 (2·3%)	30 166 (2·3%)
	American Indian or Alaska Native	5685 (0·4%)	4780 (0·4%)	4671 (0·4%)
	Native Hawaiian or other Pacific Islander	2431 (0·2%)	1835 (0·1%)	1791 (0·1%)
	Unknown	359 849 (24·2%)	298 536 (23·2%)	299 316 (23·3%)
Ethnicity				
	Hispanic or Latino	189 622 (12·7%)	146 593 (11·4%)	146 910 (11·4%)
	Not Hispanic or Latino	947 086 (63·7%)	834 868 (65·0%)	830 486 (64·7%)
	Unknown	351 004 (23·6%)	302 976 (23·6%)	307 041 (23·9%)
Comorbidities				
	Overweight or obesity	320 520 (21·5%)	267 574 (20·8%)	265 006 (20·6%)
	Hypertensive disease	469 519 (31·6%)	392 616 (30·6%)	388 894 (30·3%)
	Type 2 diabetes	238 094 (16·0%)	186 867 (14·5%)	186 048 (14·5%)
Chronic lower respiratory diseases				
	Asthma	190 561 (12·8%)	179 381 (14·0%)	180 792 (14·1%)
	Bronchitis, not specified as acute or chronic	85 358 (5·7%)	81 692 (6·4%)	84 159 (6·6%)
	Other chronic obstructive pulmonary disease	77 183 (5·2%)	73 255 (5·7%)	75 799 (5·9%)
Nicotine dependence		153 651 (10·3%)	143 014 (11·1%)	144 603 (11·3%)
Psychiatric comorbidities				
	Anxiety disorders	337 877 (22·7%)	315 075 (24·5%)	314 335 (24·5%)
	Substance use disorders	208 249 (14·0%)	191 590 (14·9%)	192 580 (15·0%)
	Mood disorders	260 720 (17·5%)	240 583 (18·7%)	240 747 (18·7%)
Heart disease				
	Ischemic heart diseases	149 630 (10·1%)	127 137 (9·9%)	127 219 (9·9%)
	Other forms of heart disease	285 499 (19·2%)	245 389 (19·1%)	244 229 (19·0%)
Chronic kidney disease		112 982 (7·6%)	94 726 (7·4%)	94 474 (7·4%)
Neoplasms (benign or malignant)		302 578 (20·3%)	275 015 (21·4%)	274 985 (21·4%)
Medications				
	Antidepressants	342 395 (23·0%)	318 657 (24·8%)	318 406 (24·8%)
	Antipsychotics	110 197 (7·4%)	97 337 (7·6%)	97 186 (7·6%)

Data are mean (SD) and n (%). For clarity purposes, apart from race, only characteristics with a prevalence over 5% in the unmatched COVID-19 cohort are shown here; the same table with all characteristics included is presented in the appendix (pp 15–17).

**Figure 1:**
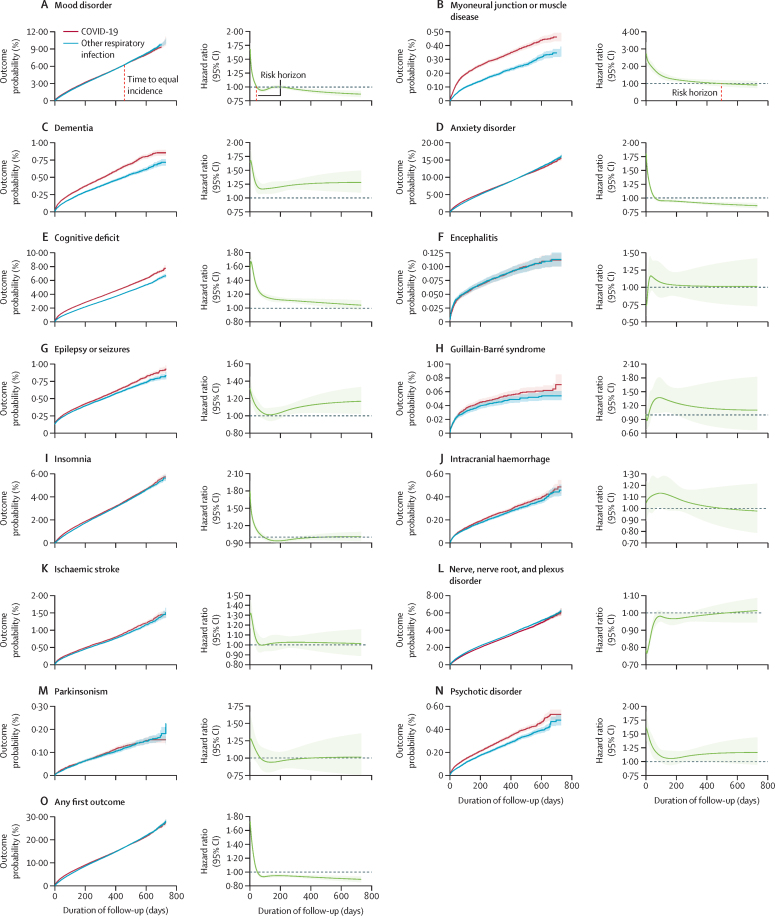
Kaplan-Meier curves and time-varying HRs over the 2-year follow-up period for each outcome (A-N) and any first outcome (O) after COVID-19 or another respiratory infection in the propensity-score matched cohorts Risk horizons are shown for panels A and B and time to equal incidence is shown on panel A; risk horizons and time to equal incidence for all other outcomes are shown in table 2. Shaded areas around curves show 95% CIs.

The 2-year risk trajectories for each outcome, and for any first outcome, are shown in figure 1 and summarised in table 2 in terms of three key statistics: HR at 6-months, risk horizon, and time to equal incidence. In terms of 6-months HRs, compared with patients with other respiratory infections, patients diagnosed with COVID-19 were at increased risk of having any first neurological or psychiatric diagnosis, and of being diagnosed with an anxiety disorder, mood disorder, psychotic disorder, insomnia, cognitive deficit, dementia, epilepsy or seizures, ischaemic stroke, intracranial haemorrhage, and myoneural junction or muscle disease, but not encephalitis, Guillain-Barré syndrome, or parkinsonism (table 2). They were at a significantly lower risk of nerve, nerve root, and plexus disorder (table 2). All 6-month HRs, p values (including Bonferroni-corrected p values), death rates, markers of severity of the index infections, and results including death as a composite outcome are shown in the appendix (pp 27–28).

**Table 2:** Risk of neurological and psychiatric sequelae at 6 months, risk horizon, and time to equal incidence for each diagnosis after COVID-19 versus after other respiratory infections, in the propensity-score matched population

	**Hazard ratio (95% CI)**	**p value**	**Risk horizon (days)**	**Time to equal incidence (days)**
Anxiety disorder	1·13 (1·11–1·15)	<0·0001	58	417
Cognitive deficit	1·36 (1·33–1·39)	<0·0001	NR	NR
Dementia	1·33 (1·26–1·41)	<0·0001	NR	NR
Encephalitis	0·96 (0·85–1·08)	0·50	..	..
Epilepsy or seizures	1·14 (1·09–1·19)	<0·0001	NR	NR
Guillain-Barré syndrome	1·12 (0·97–1·30)	0·12	..	..
Insomnia	1·13 (1·10–1·16)	<0·0001	90	NR
Intracranial haemorrhage	1·09 (1·01–1·18)	0·020	506	658
Ischaemic stroke	1·11 (1·06–1·17)	<0·0001	66	712
Mood disorder	1·08 (1·06–1·11)	<0·0001	43	457
Myoneural junction or muscle disease	1·89 (1·76–2·04)	<0·0001	497	NR
Nerve, nerve root, and plexus disorder	0·89 (0·87–0·91)	<0·0001	..	..
Parkinsonism	1·04 (0·92–1·17)	0·58	..	..
Psychotic disorder	1·27 (1·18–1·37)	<0·0001	NR	NR
Any first outcome	1·13 (1·11–1·15)	<0·0001	48	469

The risk horizon is the time at which the time-varying hazard ratio returns to 1 (ie, the baseline risk in the comparison cohort). The time to equal incidence is the time at which the cumulative incidences of the two cohorts become equal. The risk horizon and time to equal incidence are only included for outcomes with a significantly increased hazard ratio at 6 months; for outcomes that did not reach the risk horizon or time to equal incidence within the follow-up period (up to 730 days), they are shown as not reached (NR).

**Figure 2:**
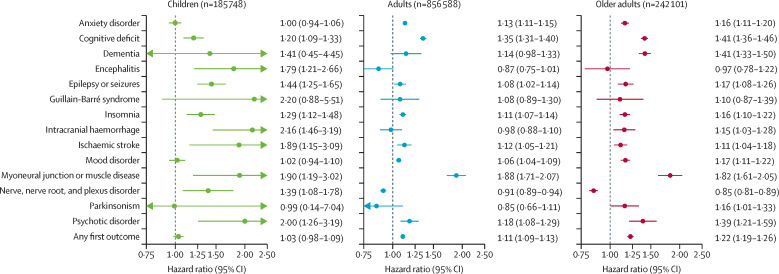
Hazard ratios for the 6-month risk of neurological and psychiatric sequelae after COVID-19 versus another respiratory infection, in different age groups, in the propensity-score matched population Data are hazard ratios with 95% CIs. Children defined as younger than 18 years, adult, aged 18–64 years, and older adults as aged 65 years or older

The results for children, adults and older adults are presented in the appendix (pp 12–14, 39–31). In terms of HRs at 6 months, four main differences from the whole cohort were observed (figure 2). First, in children, unlike older groups, there was no increased risk of mood or anxiety disorder. Second, children, unlike other age groups, were at an increased risk of encephalitis after COVID-19 compared with other respiratory infection. Third, unlike adults and older adults who were at a reduced risk of nerve, nerve root, and plexus disorder compared with the matched cohort of patients with another respiratory infection, children were at an increased risk of this outcome after COVID-19. Fourth, the risk of any first diagnosis was higher in older adults than in younger age groups, reflecting their higher HRs for most individual diagnoses.

Besides the 6-months HRs, 2-year risk trajectories can be summarised by their risk horizon and time to equal incidence. In terms of these two statistics, outcomes fell into three categories (figure 1, table 2; see appendix pp 12–14, 29–31 for a breakdown by age groups). In a first category (figure 1A), within 2 years, HRs have returned to baseline (eg, mood disorder at 43 days, anxiety disorder at 58 days, and ischaemic stroke at 66 days) and an equal cumulative incidence between cohorts was subsequently reached (eg, mood disorder at 457 days, anxiety disorder at 417 days, ischaemic stroke at 712 days). Therefore, these outcomes showed a transient risk trajectory, because no increase in cumulative incidence was observed at 2 years despite an increased risk at 6 months. In a second category (figure 1B), a risk horizon was reached within 2 years but equal incidence was not reached. These outcomes showed a persistent risk trajectory: an increased cumulative incidence persists after 2 years in the COVID-19 cohort, but there is no increased risk of new diagnoses past the risk horizon. In a third category (figure 1C), which includes cognitive deficit, dementia, psychotic disorders, and epilepsy or seizures, HRs remained greater than 1 at the end of the follow-up period. These outcomes followed an ongoing risk trajectory, wherein new diagnoses were still being made more frequently after COVID-19 diagnosis than after a diagnosis of another respiratory infection up to 2 years after the index event. These different risk trajectories were broadly similar in children, adults, and older adults (appendix pp 12–14, 29–31), with three exceptions. In children, cognitive deficit followed a transient risk trajectory, with a finite risk horizon (75 days) and a finite time to equal incidence (491 days). Conversely, intracranial haemorrhage and nerve, nerve root, and plexus disorder had an ongoing risk trajectory in children, such that they did not reach a risk horizon (nor therefore a time to equal incidence) within 2 years.

**Figure 3:**
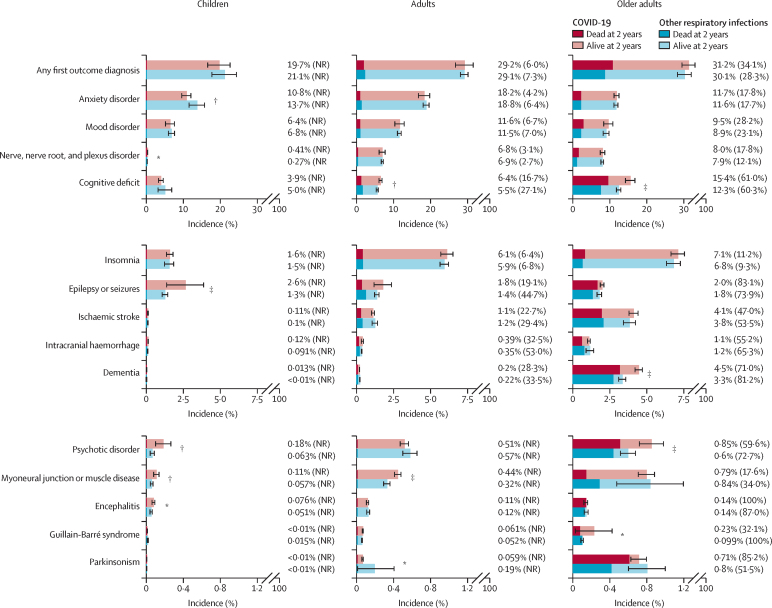
Cumulative incidence of neurological and psychiatric diagnoses at 2 years after COVID-19 versus another respiratory infection, in different age groups, by mortality status at 2 years (or censorship date), in the matched cohorts The proportion next to each bar corresponds to the overall incidence of the outcome within that age group and the number in brackets indicates the proportion of those with the outcome who died within 2 years. Estimated numbers of deaths that are lower than 120 are unreliable and therefore not reported. 95% CIs for each estimate, and p values for each outcome, are in the appendix (p 32). NR=not reported *p<0·05. †p<0·01. ‡p<0·001.

The endpoints of the 2-year risk trajectories can be summarised in terms of cumulative incidence at the end of the 2-year follow-up period and the proportion of patients with each diagnosis who subsequently died during the follow-up (figure 3; appendix pp 32–33). Interpretation of these findings, and the absence of differences between the matched cohorts with COVID-19 and other respiratory infection, should take into account the broadening 95% CI towards later stages of follow-up (as shown in the Kaplan-Meier curves in figure 1), due to the decreasing number of people contributing data. We found no evidence of a greater overall risk of any first neurological or psychiatric diagnosis after COVID-19 than after any other respiratory infection. This finding reflects the large contribution of mood and anxiety disorders to any first diagnosis, and their time to equal incidence. In older adults, death was common in those who received a neurological or psychiatric diagnosis regardless of whether they had COVID-19 or another respiratory infection, exceeding 50% for several of the neurological disorders and for psychotic disorder. The significantly higher 2-year cumulative incidences observed after COVID-19 (*vs* another respiratory infection) for outcomes with persistent or ongoing risk trajectories were reflected differently across age groups: the difference in incidence of cognitive deficit and dementia between cohorts was more noticeable for older adults than for adults or children; that of myoneural junction or muscle diseases was predominantly seen in children and adults but not older adults; that of epilepsy or seizures was significant only in children, and the risk of psychotic disorder was more evident for children and older adults than for adults.

**Figure 4:**
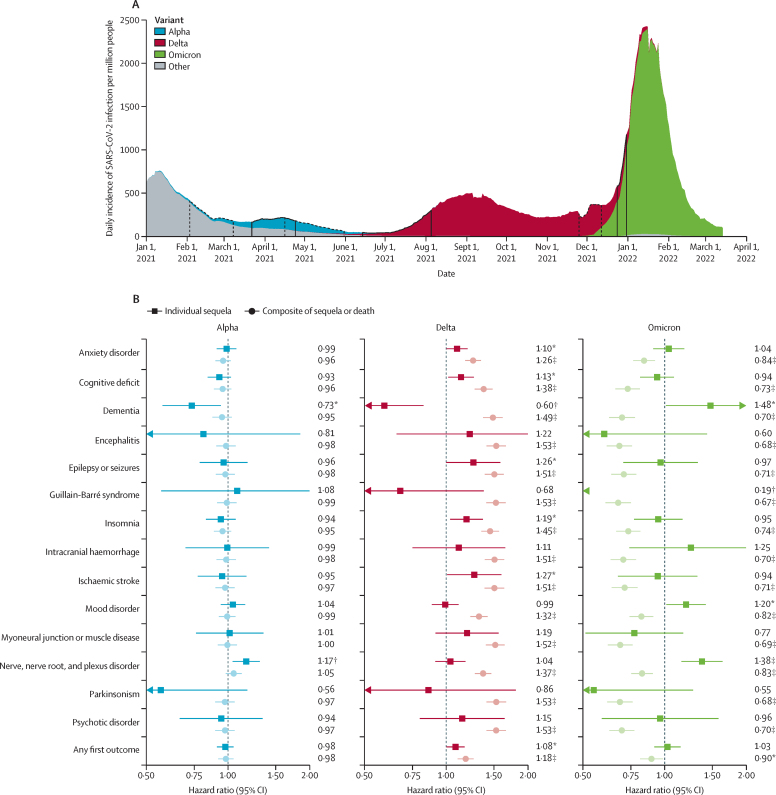
Risk of neurological and psychiatric outcomes after versus before the emergence of different SARS-CoV-2 variants, in the USA (A) Daily incidence of SARS-CoV-2 infection per million people in the USA (rolling 7-day average), by dominance of different variant. Areas surrounded by solid black lines indicate the time during which the cohort for a particular variant had their COVID-19 diagnosis. Areas surrounded by dotted black lines indicate the time during which the preceding variant cohort had their COVID-19 diagnosis. (B) Hazard ratios for the 6-month risk (140-days risk for omicron) of neurological and psychiatric sequelae, either analysed in isolation or as composite outcomes with death (ie, outcome or death). Hazard ratios with their 95% CIs and p values for each outcome, are in the appendix (pp 43–46) *p<0·05. †p<0·01. ‡p<0·001.

For our analysis of risk of outcomes with new variants, we compiled six additional US COVID-19 cohorts: the primary alpha (n=47 675), delta (n=44 835), and omicron (n=39 845) cohorts, and matched control cohorts of equal size diagnosed just before the emergence of alpha, delta, and omicron, respectively (baseline characteristics for each cohort are shown in the appendix [pp 34–42]). We found the risk profiles for each outcome evolved as new variants emerged (figure 4; appendix pp 43–46). We observed little change in terms of 6-month HRs between the patients diagnosed just before and just after the emergence of the alpha variant. By contrast, significantly higher 6-month risks of anxiety disorders, insomnia, cognitive deficit, epilepsy or seizures, and ischaemic strokes, but a lower risk of dementia, were observed in those diagnosed just after the emergence of the delta variant than in those diagnosed just before. These risks were compounded by a higher risk of death just after the emergence of the delta variant, such that the risk of composite outcomes of individual sequelae or death was all significantly higher for those diagnosed just after emergence of the delta variant (including the composite of dementia or death). Patients diagnosed with COVID-19 just after (*vs* just before) the emergence of the omicron variant were at an increased risk (over 140 days of follow-up) of dementia, mood disorders, and nerve, nerve root, and plexus disorders, and at a broadly similar risk of most other outcomes. However, risks were more than offset by a substantially lower risk of death, such that the composite risks of each outcome and death were significantly lower than among those diagnosed just before the emergence of omicron.

## Discussion

In this analysis of retrospective cohort studies, in addition to supporting previous findings of an increased risk of a range of neurological and psychiatric diagnoses in the first 6 months after COVID-19 diagnosis,^3^, ^4^, ^5^ we found substantial differences in the trajectories of these risks within the first 2 years after diagnosis. We also found that risk profiles and trajectories vary in children compared with adults and older adults, and differ between variants of SARS-CoV-2.

We summarised risk trajectories using risk horizons and times to equal incidences and our findings are of interest to both patients and clinicians. For instance, from the risk horizons, if no anxiety disorder has been diagnosed within 2 months of a COVID-19 diagnosis then, from that time onwards, a patient can be reassured that their risk is no longer any greater than after another respiratory infection. If a patient had developed an ischaemic stroke within 2 months of a COVID-19 diagnosis, it is plausible that the COVID-19 diagnosis contributed (whether directly or indirectly) to its occurrence, but beyond 2 months, other causes should be actively considered. Risk trajectories are also informative for public health. An increase in the number of new cases of COVID-19 is likely to lead to an increase in the number of cases of mood and anxiety disorders but this will be short lived. By contrast, the absence of risk horizons within the first 2 years of a COVID-19 diagnosis (ie, ongoing risk trajectories) for some diagnoses (eg, psychotic disorders, epilepsy or seizures, cognitive deficit, and dementia) indicate that patients and clinicians must remain vigilant about the possibility of these delayed sequelae. These findings also suggest that service provision needs to be reinforced and sustained, because new cases are likely to occur for a considerable time after the pandemic has subsided. The time to equal incidence informs us about what happens after the risk horizon has been reached. On the one hand, the risks might become approximately equal in the two cohorts so that a so-called COVID excess remains throughout follow-up and the time to equal incidence is never reached (ie, persistent risk trajectories), which was the case for insomnia and myoneural junction or muscle disease. On the other hand, the risks might reverse, with more new diagnoses in the other respiratory infection cohort than in the COVID-19 cohort after the risk horizon, so that a time to equal incidence is eventually reached (ie, transient risk trajectories), as seen for anxiety and mood disorders.

Another important aspect of outcome trajectories is the proportion of people who received a neurological or psychiatric diagnosis who subsequently died. All-cause mortality was substantial among older adults diagnosed with neurological or psychiatric sequelae both after COVID-19 diagnosis and after another respiratory infection—notably, those with epilepsy or seizures, dementia, cognitive deficit, and psychotic disorder. The fact that similar proportions of patients with these outcomes died in both cohorts suggests that this high mortality reflects general physical ill health rather than being related to SARS-CoV-2 infection itself.

The mortality rate in older patients also raises the issue of death as a competing risk.^22^ Because both death and the individual outcomes tend to be more common after COVID-19, the survivorship bias introduced when analysing individual outcomes brings HRs closer to 1. Individual outcomes, rather than composite outcomes with death, better reflect the burden of post-COVID-19 sequelae on health systems whereas composite outcomes are probably more informative to patients. Some outcomes have an HR of less than 1 when analysed in isolation and an HR of more than 1 when investigated as part of a composite outcome with death. Outcomes in this category are less likely to occur after COVID-19 versus after any other respiratory infection, but this might at least partly be because patients died before they could be diagnosed with these outcomes. The role of death as a competing risk likely differs between age groups because death rates vary substantially, which might contribute to apparent differences in risk profiles.

Compared with adults and older adults, children were at a particularly increased risk of epilepsy or seizures, encephalitis, and nerve, nerve root, and plexus disorder, leading to significantly higher cumulative incidence after 2 years (albeit with small absolute risks) in this age group. The persistence and severity of these outcomes cannot be determined from our study, but some will probably have deleterious consequences for children's health and physical and educational development. Therefore, these findings inform the risks and benefits of vaccination (and other preventive measures) against COVID-19 in paediatric populations. Reassuringly, unlike adults, children were not at an increased risk of mood and anxiety disorders after SARS-CoV-2 infection (even in the first 6 months) and cognitive deficit in children had a transient risk trajectory rather than ongoing risks as seen in older groups. The difference in profiles and trajectories of risks in children might indicate that the pathogenesis of COVID-19 sequelae is different in some respects from that of adults.

The risk of neurological and psychiatric diagnoses of COVID-19 was greater with the emergence of the delta variant (eg, for cognitive deficit, epilepsy or seizures, and ischaemic strokes) than just before its emergence. These risks were compounded by an increased risk of death (consistent with existing literature^23^)—for example, the HR for the composite of death or cognitive deficit was 1·38 (95% CI 1·27–1·48) whereas the HR for a diagnosis of cognitive deficit alone was 1·13 (1·02–1·26). Compared with just before the emergence of omicron, the neurological and psychiatric profile just after the emergence of omicron was broadly similar. For instance, we found no difference in the risk of cognitive deficit, epilepsy or seizures, ischaemic stroke, and psychotic disorder, and higher risks of some outcomes (eg mood disorder). All risks were largely offset by a reduced risk of death after the emergence of omicron (consistent with existing literature^13^). The decreased composite risks of death and neurological or psychiatric sequelae are reassuring for patients. However, the ongoing risk of individual outcomes indicates that health services will likely continue to face a similar rate of these post-COVID-19 diagnoses even with SARS-CoV-2 variants that lead to otherwise less severe disease.

The possible mechanisms underlying neurological and psychiatric consequences of COVID-19 remain to be determined in longitudinal and multifaceted studies.^24^, ^25^, ^26^ Sequelae in children might in part be driven by a post-infectious immune-mediated mechanism such as acute disseminated encephalomyelitis (ADEM), as has been suggested in a prospective study of 52 children hospitalised with COVID-19.^9^ This is consistent with our observations of an increased risk of encephalitis in children only, and a higher rate of post-COVID epilepsy or seizures in children. In the whole cohort, the finding of a persisting increased risk of cognitive deficit and dementia, psychotic disorder, and epilepsy or seizures 2 years after SARS-CoV-2 infection suggests that any underlying mechanism must have ongoing activity well past the acute infection (e.g. endotheliopathy might lead to a damaged or fragile cerebral vasculature at risk of thrombotic events or recurrent leakage^27^). Notably, mood and anxiety disorders followed a different pattern than most other outcomes: their elevated risk subsided within 2 months, their cumulative incidence after 2 years was not increased, and children were not at greater risk at any stage after COVID-19 than after other respiratory infections. One possible explanation is that COVID-19 precipitates mood and anxiety disorders in individuals with an underlying predisposition, via a short-lived stress-related pathogenesis to which children are less susceptible.

Our study has specific limitations in addition to those inherent in electronic health records studies. First, our COVID-19 cohorts are probably enriched for symptomatic cases because self-diagnosed or asymptomatic COVID-19 is less likely to be coded in the health record. This is also true of the comparator cohort and their respiratory infections, and so HRs are less affected by this limitation than are incidences. Second, COVID-19 appeared to be more severe than other respiratory infections, but the mediation of our results by severity of the illness was not analysed. However, mediation by several markers of severity has been tested in our previous study,^4^ in which we showed that severity explains part, but not all, of the association between COVID-19 and specific neurological and psychiatric outcomes. Third, only individuals who were diagnosed early in the pandemic contributed data for the whole 2-year follow-up. This is a subgroup within the whole cohort that might not be representative of the whole cohort. Future studies should clarify risks at the 2-year time point once larger sample sizes with longer follow-up become available. Fourth, our allocation of cohorts to study variants is based on epidemiological incidence data of different variants, not individual genotyping. Hence, these cohorts are likely to contain a few cases of other variants, which we factored into the statistical power calculation. The presence of patients with different variants in each variant cohort will bring HRs closer to 1 and so differences between variants would likely be more substantial if individual genotyping were possible. Fifth, vaccination status (used in matching) is probably under-reported in TriNetX, because the prevalence of vaccination was low in both cohorts. This under-reporting might affect HRs calculated when comparing COVID-19 cohorts before and after the emergence of new variants. Selecting time windows that were close to each other mitigates this effect, but does not eliminate it. Previous vaccination is associated with reduced or unchanged risks of most neurological or psychiatric outcomes.^28^ Therefore, the higher number of vaccinated people after (*vs* before) the emergence of each variant might have decreased the observed HRs. Sixth, children and adolescents were grouped together, so further studies are needed to characterise the risks in different paediatric subgroups. Seventh, although in-hospital mortality data are well captured in TriNetX, out-of-hospital mortality reporting is more variable and linkage with mortality indices is only partial, so our incidence estimates will be underestimates and should be interpreted cautiously; but HRs for composite outcomes should not be affected to the same extent. Eighth, we do not know the severity or course of each disorder after diagnosis, or whether or not these are similar after COVID-19 and after other respiratory infections.

In summary, post-COVID neurological and psychiatric outcomes followed different risk trajectories: the risk of cognitive deficit, dementia, psychotic disorder, and epilepsy or seizures remained increased at 2 years after a COVID-19 diagnosis, while the risks of other diagnoses (notably, mood and anxiety disorders) subsided early and showed no overall excess over the 2-year follow-up. Children are not at increased risk of mood or anxiety disorders (even over the first 6 months) but share adults’ risk of several other diagnoses. The comparable risks seen after the emergence of omicron indicate that the neurological and psychiatric burden of COVID-19 might continue even with variants that lead to otherwise less severe disease. These findings are relevant for policy makers involved in anticipating and addressing the health burden of the pandemic, for researchers seeking to identify the mechanisms underpinning brain sequelae of COVID-19, and for patients and clinicians wishing to know the neurological and psychiatric risks following SARS-CoV-2 infections.

## Data sharing

The TriNetX system returned the results of these analyses as csv files, which we downloaded and archived. Aggregate data, as presented in this Article, can be freely accessed at https://osf.io/snfhw. The data used for this Article were acquired from TriNetX. This study had no special privileges. Inclusion criteria specified in the Methods and appendix would allow other researchers to identify similar cohorts of patients as we used here for these analyses; however, TriNetX is a live platform with new data being added daily so exact counts will vary. To gain access to the data, a request can be made to TriNetX (join@trinetx.com), but costs might be incurred, and a data sharing agreement would be necessary.

For more on **US Centers for Disease Control and Prevention COVID data tracker** see https://covid.cdc.gov

For more on **TriNetX website** see https://www.trinetx.com/platform

For more on **aggregate data** see https://osf.io/snfhw

For more on **TriNetX website** see https://www.trinetx.com/platform

## Declaration of interests

We declare no competing interests.
