# Supplementary material for: Neurological and psychiatric risk trajectories after SARS-CoV-2 infection: an analysis of 2-year retrospective cohort studies including 1 284 437 patients
Source: Lancet Psychiatry. 2022 Oct;9(10):815–27. doi: 10.1016/S2215-0366(22)00260-7 (PMC9385200; doi:10.1016/S2215-0366(22)00260-7)
Supplement: Supplementary appendix [file mmc1.pdf]

THE LANCET
Psychiatry

Supplementary appendix

This appendix formed part of the original submission and has been peer reviewed. We post it as supplied by the authors.

Supplement to: Taquet M, Sillett R, Zhu L, et al. Neurological and psychiatric risk trajectories after SARS-CoV-2 infection: an analysis of 2-year retrospective cohort studies including 1 284 437 patients. *Lancet Psychiatry* 2022; published online Aug 17. https://doi.org/10.1016/S2215-0366(22)00260-7.

Supplementary Methods

TriNetX network

This section largely replicates our previous description of the network.[1]

Legal and ethical status
TriNetX's networks are compliant with the Health Insurance Portability and Accountability Act (HIPAA), the US federal law which protects the privacy and security of healthcare data. TriNetX is certified to the ISO 27001:2013 standard and maintains an Information Security Management System (ISMS) to ensure the protection of the healthcare data it has access to and to meet the requirements of the HIPAA Security Rule. Any data displayed on the TriNetX Platform in aggregate form, or any patient level data provided in a data set generated by the TriNetX Platform, only contains de-identified data as per the de-identification standard defined in Section §164.514(a) of the HIPAA Privacy Rule. The process by which the data is de-identified is attested to through a formal determination by a qualified expert as defined in Section §164.514(b)(1) of the HIPAA Privacy Rule. This formal determination by a qualified expert, refreshed in December 2020, supersedes the need for TriNetX's previous waiver from the Western Institutional Review Board (IRB). The network contains data that are provided by participating Health Care Organizations (HCOs), each of which represents and warrants that it has all necessary rights, consents, approvals and authority to provide the data to TriNetX under a Business Associate Agreement (BAA), so long as their name remains anonymous as a data source and their data are utilized for research purposes. The data shared through the TriNetX Platform are attenuated to ensure that they do not include sufficient information to facilitate the determination of which HCO contributed which specific information about a patient. The HCOs warrant that they have all necessary rights, consents, approvals, and authority to provide the data to TriNetX, as long as their name remains anonymous as a data source and their data are used for research purposes. Keeping the identity of participating HCOs from the researchers using the data also contribute to complying with legal frameworks and ethical guidelines guarding against data re-identification.

Acquisition of data, quality control, and other procedures
The data are stored onboard a TriNetX appliance – a physical server residing at the institution's data centre or a virtual hosted appliance. The TriNetX platform is a fleet of these appliances connected into a federated network able to broadcast queries to each appliance. Results are subsequently collected and aggregated.

Once the data are sent to the network, they are mapped to a standard and controlled set of clinical terminologies and undergo a data quality assessment including 'data cleaning' that rejects records which do not meet the TriNetX quality standards. HIPAA compliance of the clinical patient data is achieved using de-identification. Different data modalities are available in the network. They include demographics (coded to HL7 version 3 administrative standards), diagnoses (represented by ICD-10-CM codes), procedures (coded in ICD-10-PCS or CPT), and measurements (coded to LOINC). While extensive information is provided about patients' diagnoses and procedures, other variables (such as socioeconomic and lifetime factors are not comprehensively represented).

The data from a typical HCO generally go back around 7 years, with some going back 13 years. The data are continuously updated. HCOs update their data at various times, with most refreshing every 1, 2, or 4 weeks. The data for patients diagnosed with COVID-19 come primarily (>98%) from HCOs in the USA, with the remainder coming from Australia, UK, Spain, Bulgaria, India, Malaysia, and Taiwan.

Data quality assessment followed a standardised strategy wherein the data are reviewed for conformance (adherence to specified standards and formats), completeness (quantifying data presence or absence) and plausibility (believability of the data from a clinical perspective). There are pre-defined metrics for each of the above assessment categories. Results for these metrics are visualised and reviewed for each new site that joins the network as well as on an ongoing basis. Any identified issue is communicated to the data provider and resolved before continuing data collection.

The basic formatting of contributed data is also checked (e.g. to ensure that dates are properly represented). Records are checked against a list of required fields (e.g., patient identifier) and rejects those records for which the required information is missing. Referential integrity checking is done to ensure that data spanning multiple database tables can be successfully joined together. As the data are refreshed, changes in volume of data over

time is monitored to ensure data validity. At least one non-demographic fact for each patient is required for them to be counted in the dataset. Patient records with only demographics information are discarded.

The software also undergoes quality control. The engineers testing the software are independent from the engineers developing it. Each test code is checked by two independent testing engineers. Each piece of software is tested extensively against a range of synthetic data (i.e. generated for the purpose of testing) for which the expected output is established independently. If the software fails to return this output, then the software is deemed to have failed the test and is examined and modified accordingly. For statistical software (including that used for propensity score matching, for Kaplan-Meier analysis, etc), an additional quality control step is implemented. Two independent codes are written in two different programming languages (typically R and python) and the statistical results are compared. If discrepancies are identified, then the codes are deemed to have failed the test and are examined and modified accordingly. All the code is reviewed independently by another engineer.

The test strategy follows three levels of granularity:

1. Unit tests: These test specific blocks, or units, of code that perform specific actions (e.g. querying the database).
2. Integration tests: These ensure that different components are working together correctly.
3. End-to-end tests: These tests run the entire system and check the final output.

Some comments on advantages and disadvantages of EHR data

One advantage of EHR data, like those in TriNetX, over insurance claim data is that both insured and uninsured patients are included. An advantage of EHR data over survey data is that they represent the diagnostic rates in the population presenting to healthcare facilities. This provides an accurate account of the burden of specific diagnoses on healthcare systems. However, there are also limitations inherent to research of this kind using electronic health records,[2–4] including TriNetX:

1. Undiagnosed patients who might have COVID-19 but did not seek medical attention (or in whom the diagnosis was missed) are not included.
2. Despite the matching and use of various comparison cohorts, there may well be residual confounding, particularly related to social and economic factors which are not well captured in EHR networks and which might influence outcomes post COVID-19.
3. We do not know which diagnoses were made in primary or secondary care or specialist facilities, nor by whom.
4. A patient may be seen in different HCOs for different parts of their care, and if one HCO is not part of the federated network then part of their medical records may not be available. Using a network of HCOs (rather than a single HCO) limits this possibility but does not fully eliminate it.
5. The severity and persistence of diagnoses is difficult to assess using EHR data as this is not typically coded. As a result, we can comment on incidence of new cases but cannot assess the duration of illness.
6. Since the data are presented as they are recorded, we cannot be sure that there has not been mis-recording of information, adding a degree of noise to the data.
7. Historical data before the start of EHRs (or the addition of an HCO to the network) may well be incomplete. For example, a previous psychiatric or neurological diagnosis may not have been recorded.

Differences from our previous study

Our study design follows a similar approach to our previous study on the neurological and psychiatric outcomes of COVID-19 using the same EHR network.[5] However, there are some key differences which mean that results cannot be directly compared between the two studies and apparent discrepancies might occur. We summarise them here.

- **Children**: We included children of all ages in our cohort whereas in our previous study children under 10 were excluded. This was motivated by our interest in assessing the risk profile in children diagnosed with COVID-19.

- **Vaccinated individuals**: At the time of our previous study, very few people had been vaccinated against COVID-19 and so those results reflected the sequelae among unvaccinated individuals. In the present study, we included all individuals diagnosed with COVID-19 regardless of vaccination status.

In the later stages of ‘recruitment’, many of them had probably been vaccinated against COVID-19 and had post-vaccine (‘breakthrough’) infections. This affects the risks of many neurological and psychiatric outcomes.[6]

- **Control health event**: In the previous study we used other respiratory infections, and influenza, as two separate control health events. In this study, we only use other respiratory infections as a control health event given that (i) the number of people diagnosed with influenza is highly influenced by the season and so our findings would only be representative of people diagnosed with COVID-19 during the influenza season (since the analysis is now stratified by date of the index event), and (ii) the influenza cohort has become much smaller than the COVID-19 cohort so that after 1:1 matching, many patients with COVID-19 would have been excluded and those who were included would have had baseline characteristics resembling those of the influenza cohort and so lack generalizability to patients at very low risk of influenza. By contrast, the cohort of patients diagnosed with another respiratory infection vastly outnumbers the COVID-19 cohort so that after 1:1 matching, the matched COVID-19 cohort contains a large majority of the whole COVID-19 cohort (86% as can be seen in Table 1) and its baseline characteristics are similar to those of the whole COVID-19 cohort.

- **Outcomes**: We added two outcomes to the list that we investigated in our previous study: cognitive deficit (designed to capture post-COVID ‘brain fog’, using the same definition in terms of ICD-10 codes as in another study[1]) and epilepsy or seizures given evidence of acute seizures[7] and electroencephalographic changes[8] in patients with COVID-19, and our recent 6-months study.[9] We removed substance use disorders from the list of outcomes of interest for two reasons: (i) it includes as one of its most prevalent subcategories “Nicotine dependence” (ICD-10 code F17) which we suspect might be coded preferentially in people with another respiratory infection (e.g. after a diagnosis of COPD), and (ii) it is a common diagnosis and therefore might drive the composite endpoint of “any first” diagnosis which, given our first concern, might itself become unreliable.

- **Survivors and non-survivors**: In our previous study, we had limited our analysis to patients who were still alive at the time of the analysis. In this study, we included both patients who were still alive and patients who had died. Three factors motivated this change: (i) it allowed us to gain insight into the clinical trajectory of patients (as presented in Fig. 3), (ii) it better represents the overall burden of neuropsychiatric sequelae of COVID-19 by including all patients diagnosed with COVID-19, and (iii) because the follow-up is up to 2 years, a substantial minority of people (in particular in the older adults cohort) die (of any cause) during the follow-up and excluding them would reduce generalizability of the findings.

- **Stratification by date of index event**: In this study, we stratified the analysis by date of the index event which provides better control for contextual factors that varied during the pandemic (e.g. lockdown in place or not, school open or not, etc).

Definition of cohorts

The control cohort consisted of patients with a diagnosis of any respiratory infection other than COVID-19 (J00–06, J09–18, or J20–22), specifically, those with any of the following diagnoses:

J00: Acute nasopharyngitis
J01: Acute sinusitis
J02: Acute pharyngitis
J03: Acute tonsillitis
J04: Acute laryngitis and tracheitis
J05: Acute obstructive laryngitis [croup] and epiglottitis
J06: Acute upper respiratory infections of multiple and unspecified sites
J09: Influenza due to certain identified influenza viruses
J10: Influenza due to other identified influenza virus
J11: Influenza due to unidentified influenza virus
J12: Viral pneumonia, not elsewhere classified
J13: Pneumonia due to Streptococcus pneumoniae
J14: Pneumonia due to Hemophilus influenzae
J15: Bacterial pneumonia, not elsewhere classified

J16: Pneumonia due to other infectious organisms, not elsewhere classified
J17: Pneumonia in diseases classified elsewhere 3
J18: Pneumonia, unspecified organism
J20: Acute bronchitis
J21: Acute bronchiolitis
J22: Unspecified acute lower respiratory infection

Because some patients with the control index event may have had COVID-19 at a different point in time, we excluded from the control cohorts all those who had COVID-19 diagnosed at any point in time. To avoid any contamination between cohorts, COVID-19 as an exclusion criterion was defined in the broader sense to be all patients with a confirmed diagnosis of COVID-19 (ICD-10 code U07.1) but also patients with an unconfirmed COVID-19 diagnosis (U07.2), a recorded positive PCR test for COVID-19, or any of the following recorded on or after January 20, 2020: Pneumonia due to SARS-associated coronavirus (J12.81), Other coronavirus as the cause of disease classified elsewhere (B97.29), or Coronavirus infection unspecified (B34.2). Inclusion of the latter three diagnostic codes captures patients who receive a COVID-19 diagnosis in the early stage of the pandemic when the ICD code for COVID-19 (U07) was not yet defined. Specifically, the following codes were excluded from the control cohorts if they occurred on or after January 20, 2020:

U07.1: COVID-19, virus identified
U07.2: COVID-19, virus not identified
J12.81: Pneumonia due to SARS-associated coronavirus
B97.29: Other coronavirus as the cause of disease classified elsewhere
B34.2: Coronavirus infection, unspecified
Positive SARS-CoV-2 RNA in Respiratory specimen
Positive SARS-CoV-2 RNA in Unspecified specimen
Positive SARS-CoV-2 N gene in Respiratory specimen
Positive SARS-CoV-2 N gene in Unspecified specimen
sitive SARS-CoV-2 RdRp gene in Respiratory specimen
Positive SARS-CoV-2 E gene in Respiratory specimen
Positive SARS-CoV-2 E gene in Unspecified specimen
Positive SARS-CoV-2 RNA panel in Respiratory specimen
Positive SARS-CoV-2 RNA panel in Unspecified specimen
Positive SARS-CoV-2 RNA in Nasopharynx
Positive SARS coronavirus 2 and related RNA
Positive SARS-related coronavirus RNA in Respiratory specimen
Positive SARS coronavirus 2 ORF1ab in Respiratory specimen

Cohorts were stratified by date of the index event in 2-monthly bins (January 20 to March 19, 2020, March 20 to April 19, 2020, etc.). Matching was achieved independently within each period and age group. In order to capture contextual changes during the pandemic, the smaller the time window bins, the better. However, smaller bins mean that fewer individuals are available in the control cohort for matching and so poorer matching might be achieved. Two-monthly periods appeared to be an adequate compromise which allows to capture the dynamics of contextual changes during the pandemic.

The duration of follow-up of the patients depended on the date of their index event. Patients were all followed up until the date of main analysis on 13th April 2022, corresponding to a maximum of 27 months follow-up. The Kaplan-Meier estimator accommodates differences in duration of follow-up by means of censoring.

Additional cohorts for analysis of the risk profiles of specific variants were created, specifically for the alpha, delta and omicron variants. Cohorts were generated using the following time points (determined based on statistical power calculation; see Details on statistical analysis below), with matched control cohorts generated from time periods just before the emergence of the variant in question (incidence data to determine these time windows were extracted from OurWorldInData.org, and variant data were extracted from CoVariants.org):

- **Alpha variant**: comparing cohorts diagnosed between 22/03/2021 and 24/04/2021 (i.e. post-emergence of the alpha variant) to a cohort diagnosed between 03/02/2021 and 08/03/2021 (i.e. pre-emergence of the alpha variant)

- **Delta variant**: comparing cohorts diagnosed between 14/06/2021 and 05/08/2021 (i.e. post-emergence of the delta variant) to a cohort diagnosed between 16/04/2021 and 31/05/2021 (i.e. pre-emergence of

the delta variant)

- **Omicron variant**: comparing cohorts diagnosed between 24/12/2021 and 31/12/2021 (i.e. post-emergence of the omicron variant) to a cohort diagnosed between 25/11/2021 and 12/12/2021 (i.e. pre-emergence of the omicron variant)

For all cohorts, when investigating the incidence of a first diagnosis, those with that diagnosis recorded at any point before the index event were excluded. This was achieved on a per-outcome basis so that cohorts varied slightly between outcomes (e.g. when investigating the incidence of a first ischaemic stroke, those with an ischaemic stroke recorded before their index infection were excluded, but they could still be included when investigating the incidence of a first psychotic diagnosis).

Definition of covariates

To reduce the effect of confounding on associations between a diagnosis of COVID-19 and a subsequent neurologic or psychiatric diagnosis, cohorts were matched for established or suspected risk factors for COVID-19[10–13] and for established risk factors for COVID-19 death[14] (taken to be risk factors of a more severe COVID-19 illness). These were the covariates used in our previous studies.[1,5,6,13,15] In this study, in addition to these covariates, cohorts were matched for COVID-19 vaccination and for possible concurrent medications which are known to be associated with different incidence or outcomes of COVID-19, namely lithium,[15] antidepressants and fluvoxamine in particular,[16] and antipsychotics and clozapine in particular.[17] Because a history of the neurological and psychiatric outcomes are likely to correlate with future recurrence of these outcomes, each of the outcomes were also included as a covariate. The following confounding factors were therefore included (with ICD-10/CDC codes in brackets):

1) **Age** at the time of index event.
2) **Sex** coded as female, male, or other.
3) **Race** encoded as 6 separate dichotomous variables: White (2106-3), Black or African American (2054-5), American Indian or Alaska Native (1002-5), Asian (2028-9), Native Hawaiian or Other Pacific Islander (2076-8), or Unknown Race (2131-1).
4) **Ethnicity** encoded as Hispanic or Latino (2135-2), Not Hispanic or Latino (2186-5), or Unknown Ethnicity.
5) **Socioeconomic deprivation** encoded as the ICD-10 code for Problems related to housing and economic circumstances (Z59).
6) **Obesity** encoded as Overweight and obesity (E66)
7) **Hypertension** encoded as 2 dichotomous variables: Hypertensive diseases (I10-I16) and the now deprecated version that was used until 2018 Hypertension diseases (I10-I15).
8) **Diabetes mellitus** encoded as 2 dichotomous variables: Type 1 diabetes mellitus (E10) and Type 2 diabetes mellitus (E11).
9) **Chronic lower respiratory diseases** encoded by each sub-category of the corresponding ICD-10 group: Bronchitis, not specified as acute or chronic (J40), Simple and mucopurulent chronic bronchitis (J41), Unspecified chronic bronchitis (J42), Emphysema (J43), Other chronic obstructive pulmonary disease (J44), Asthma (J45), Bronchiectasis (J47).
10) **Nicotine dependence** encoded as the corresponding ICD-10 diagnosis (F17.2).
11) **Substance use disorders** encoded as the ICD-10 code for mental and behavioural disorders due to psychoactive substance use (F10-F19).
12) **Psychotic disorders** encoded as the ICD-10 code for schizophrenia, schizotypal, delusional, and other non-mood psychotic disorders (F20-F29).
13) **Mood disorders** encoded as a single variable (as well as individual codes, see below) with any of the ICD-10 code for mood disorders (F30-F39).
14) **Anxiety disorders** encoded as the ICD-10 code for anxiety, dissociative, stress-related, somatoform and other nonpsychotic mental disorders (F40-F48)
15) **Heart diseases** encoded as 2 categorical variables: Ischaemic heart disease (I20-I25) and Other forms of heart disease (I30-I52).
16) **Chronic kidney disease** encoded as 2 dichotomous variables: Chronic kidney disease (N18) and Hypertensive chronic kidney disease (I12).
17) **Chronic liver disease** encoded as 8 categorical variables: Alcoholic liver disease (K70), Hepatic failure, not elsewhere classified (K72), Chronic hepatitis, not elsewhere classified (K73), Fibrosis and cirrhosis of

liver (K74), Fatty (change of) liver, not elsewhere classified (K76.0), Chronic passive congestion of liver (K76.1), Portal hypertension (K76.6), Other specified diseases of liver (K76.8).

18) **Stroke** encoded as the dichotomous variable Cerebral infarction (I63) .
19) **Dementia** encoded as 6 dichotomous variables: Vascular dementia (F01), Dementia in other diseases classified elsewhere (F02), Unspecified dementia (F03), Alzheimer's disease (G30), Frontotemporal dementia (G31.0), and Dementia with Lewy bodies (G31.83).
20) **Neoplasm and haematological cancer in particular** encoded as 2 dichotomous variables: Neoplasms (C00-D49) and Malignant neoplasms of lymphoid, hematopoietic and related tissue (C81-C96).
21) **Organ transplant** encoded as 2 dichotomous variables: Renal Transplantation Procedures and Liver Transplantation Procedures.
22) **Rheumatoid arthritis** encoded as 2 dichotomous variables: Rheumatoid arthritis with rheumatoid factor (M05) and Other rheumatoid arthritis (M06).
23) **Systemic lupus erythematosus** encoded as a dichotomous variable corresponding ICD-10 code (M32).
24) **Psoriasis** encoded as a dichotomous variable corresponding ICD-10 code (L40).
25) **Disorders involving an immune mechanism** encoded as a dichotomous variable "Certain disorders involving the immune mechanism" (D80-D89).
26) **Insomnia** encoded as 2 dichotomous variables: Insomnia (G47.0) and Insomnia not due to a substance or known physiological condition (F51.0)
27) **Intracranial haemorrhage** encoded as 3 dichotomous variables: Nontraumatic subarachnoid haemorrhage (I60), Nontraumatic intracerebral haemorrhage (I61) and Other and unspecified nontraumatic intracranial haemorrhage (I62).
28) **Parkinsonism** encoded as 2 dichotomous variables: Parkinson's disease (G20) and Secondary parkinsonism (G21).
29) **Peripheral neurological disorders** encoded as 3 dichotomous variables: Guillan-Barre syndrome (G61.0), Nerve, nerve root and plexus disorders (G50-G59) and Diseases of myoneural junction and muscle (G70-G73).
30) **Encephalopathy and encephalitis** encoded as 5 dichotomous variables: Encephalopathy, unspecified (G93.40), Encephalitis, myelitis and encephalomyelitis (G04), Encephalitis, myelitis and encephalomyelitis in diseases classified elsewhere (G05), Unspecified viral encephalitis (A86) and Other specified viral encephalitis (A85.8).
31) **Cognitive impairment and mental disorders** encoded as 3 dichotomous variables: Mild cognitive impairment, so stated (G31.84), Dyslexia and other symbolic dysfunctions, not elsewhere classified (R48), Other specified mental disorders due to known physiological condition (F06.8).
32) **Altered mental state** encoded as 3 dichotomous variables: Delirium due to known physiological condition (F05), Somnolence, stupor, coma (R40), Other symptoms and signs involving cognitive functions and awareness (R41).
33) **Epilepsy and seizures** encoded as 2 dichotomous variables: Epilepsy and recurrent seizures (G40) and Convulsions, not elsewhere classified (R56).
34) **Antipsychotics** as a class, encoded as VA Class CN700
35) **Clozapine** encoded as RxNorm 2626
36) **Antidepressants** as a class, encoded as VA Class CN600
37) **Fluvoxamine** encoded as RxNorm 42355
38) **Lithium salts** encoded as VA Class CN750
39) **SARS-CoV-2 vaccination** encoded as 4 dichotomous variables:
    a) SARS-CoV-2 (covid-19) vaccine, unspecified encoded as CVX 213
    b) Pfizer's Severe acute respiratory syndrome coronavirus 2 (SARS-CoV-2) (Coronavirus disease [COVID-19]) vaccine, mRNA-LNP, spike protein, preservative free, 30 mcg/0.3mL dosage, diluent reconstituted, for intramuscular use, encoded as CPT 91300
    c) Moderna's Severe acute respiratory syndrome coronavirus 2 (SARS-CoV-2) (Coronavirus disease [COVID-19]) vaccine, mRNA-LNP, spike protein, preservative free, 100 mcg/0.5mL dosage, for intramuscular use, encoded as CPT 91301
    d) Janssen's Severe acute respiratory syndrome coronavirus 2 (SARS-CoV-2) (Coronavirus disease [COVID-19]) vaccine, DNA, spike protein, adenovirus type 26 (Ad26) vector, preservative free, 5x10^10 viral particles/0.5mL dosage, for intramuscular use, encoded as CPT 91303

Each individual code was considered a confounding factor in and of itself, so that matching was achieved for each of them individually. For instance, matching was achieved for each subcategory (and not just for the whole category) of chronic lower respiratory diseases. For variables representing diagnoses, socioeconomic deprivation, and medications, the confounder was considered present if the diagnosis was recorded at least once in a patient's health record before the index event.

Data on race, ethnicity, and sex could be recorded as unknown and this was considered as a category of its own so that little difference in the number of people with unknown race/ethnicity/sex would be present in matched cohorts. This appeared to be a better approach than excluding those with unknown race/ethnicity/sex since this might affect the generalisability of the results (e.g. by excluding individuals who prefer not to disclose their ethnicities).

The primary analysis was stratified by age group (< 18, 18-64, ≥ 65 years-old) and age was also included as a continuous covariate within each stratum, thus providing additional controlling for age.

Definition of outcomes

All outcomes were defined as a diagnosis recorded in the patient's electronic health record from the day following the index event until the end of follow-up. As mentioned in the manuscript, for chronic illnesses, only first diagnoses were counted (i.e. patients with the diagnosis before the index event were excluded from the survival analysis). For diagnoses that can recur or relapse, we separately estimated the incidence of first diagnosis and the incidence of any diagnosis, but we focus on *first* diagnosis in the main manuscript (while presenting the results for *any* diagnosis in this appendix) given that *any* diagnosis might include re-coding of previous diagnoses. For outcomes that do not tend to recur after they have resolved (e.g., Guillain-Barré syndrome), we estimated the incidence of any diagnoses.

Specifically, the following ICD-10 codes (with the ICD-10 labels in brackets) were used to define outcomes:

1) **Anxiety disorder** (first and any diagnosis): F40-F48 (Anxiety, dissociative, stress-related, somatoform and other nonpsychotic mental disorders)

2) **Cognitive deficit** (first and any diagnosis): F01 (Vascular dementia), F02 (Dementia in other diseases classified elsewhere), F03 (Unspecified dementia), F05 (Delirium due to known physiological condition), F06.8 (Other specified mental disorders due to known physiological condition), G30 (Alzheimer's disease), G31.0 (Frontotemporal dementia), G31.83 (Dementia with Lewy bodies), G31.84 (Mild cognitive impairment, so stated), G93.40 (Encephalopathy, unspecified), R40 (Somnolence, stupor and coma), R41 (Other symptoms and signs involving cognitive functions and awareness), R48 (Dyslexia and other symbolic dysfunctions, not elsewhere classified). These terms which mix actual diagnoses and symptoms were used to capture the range of diagnostic codes that patients presenting with 'brain fog' might receive. They are the same terms as used in our previous study of long-COVID features.[1] This outcome overlaps with the next one ('Dementia') by including all dementia codes. The reason for including dementia diagnoses in 'cognitive deficit' is to account for possible misdiagnoses of brain fog as dementia, and to more fully represent any cognitive deficit that can occur after COVID-19.

3) **Dementia** (first diagnosis): F01 (Vascular dementia), F02 (Dementia in other diseases classified elsewhere), F03 (Unspecified dementia), G30 (Alzheimer's disease), G31.0 (Frontotemporal dementia), G31.83 (Dementia with Lewy bodies)

4) **Encephalitis** (any diagnosis): G04 (Encephalitis, myelitis and encephalomyelitis), G05 (Encephalitis, myelitis and encephalomyelitis in diseases classified elsewhere), A86 (Unspecified viral encephalitis), or A85.8 (Other specified viral encephalitis)

5) **Epilepsy/seizures** (first and any diagnosis): G40 (Epilepsy and recurrent seizures), R56 (Convulsions, not elsewhere classified)

6) **Guillain-Barré syndrome** (any diagnosis): G61.0 (Guillain-Barré syndrome)

7) **Insomnia** (first and any diagnosis): F51.0 (Insomnia not due to a substance or known physiological condition) or G47.0 (Insomnia)

8) **Intracranial haemorrhage** (first and any diagnosis): I60 (non-traumatic subarachnoid haemorrhage), I61 (non-traumatic intracerebral haemorrhage), and I62 (other and unspecified non-traumatic intracranial haemorrhage)

9) **Ischaemic stroke** (first and any diagnosis): I63 (cerebral infarction)

10) **Mood disorder** (first and any diagnosis): F30-F39 (Mood disorders). ICD-10 codes representing remission (e.g. F32.4 - Major depressive disorder, single episode, in partial remission) were excluded.

11) **Myoneural junction/muscle disease** (first diagnosis): G70-G73 (Diseases of myoneural junction and muscle) – these disorders are often called neuromuscular, but we use the ICD-10 term

12) **Nerve/nerve root/plexus disorders** (any diagnosis): G50-G59 (Nerve, nerve root and plexus disorders)

13) **Parkinsonism** (first diagnosis): G20 (Parkinson's disease) or G21 (Secondary parkinsonism)

14) **Psychotic disorder** (first and any diagnosis): F20-F29 (Schizophrenia, schizotypal, delusional, and other non-mood psychotic disorders)

Composite outcomes with death were also recorded. TriNetX records death from EHR which accurately record in-hospital deaths as well as some out-of-hospital deaths. For a subset of patients, linkage with third-party mortality data sources (SSA, Private Obituary Data, and Private Claims Data) is used to increase coverage of out-of-hospital deaths. For privacy purposes, TriNetX never returns fewer than 10 events per query. As our analysis is stratified by date of index event (12 strata in total), the minimum number of deaths reported is 120. When the number of deaths following a neurological or psychiatric outcome falls below this threshold, it was deemed unreliable and not reported (as was the case for all outcomes in children and some outcomes in adults).

Details on statistical analyses

Implementation details of propensity score matching
In propensity score matching, the propensity score was calculated using a logistic regression (implemented by the function LogisticRegression of the scikit-learn package in Python 3.7) including each of the covariates mentioned above. To eliminate the influence of ordering of records, the order of the records in the covariate matrix were randomised before matching.

Estimating time-varying hazard ratios
Time-varying HRs were assessed using natural cubic splines (in log-time) fitted to the log-cumulative hazard.[18] This was achieved using the generalized survival models of the rstpm2 package (version 1.5.1) in R.[19] As recommended by Royston and Parmar,[18] splines with 1, 2, and 3 degrees of freedom were estimated for both the baseline log-cumulative hazard and its cohort dependency and the number of degrees of freedom leading to the lowest Akaike Information Criterion (AIC) was selected. This was achieved on a per-comparison basis so that more complex time dependency (i.e. higher number of degrees of freedom) could be selected for a specific comparison if there was enough evidence in the data to support such complexity.

Statistical power calculation and definition of time windows for the variant analysis
For the analysis of SARS-CoV-2 variants, the time windows to define cohorts were selected to achieve sufficient statistical power while keeping the intervals between the time windows short. At every point of the 'variant' time windows, the prevalence of the corresponding variant had to be at least 50% (although at most points, it was much higher than that) while in the 'control' time windows, the prevalence had to be less than 20% at every point (and at most points it was much less than that). Incidence data were extracted from OurWorldInData.org, and variant data were extracted from CoVariants.org.

Statistical power was estimated using Freedman's method for log-rank tests[20] and implemented in the powerCT function of the powerSurvEpi package (version 0.1.3) in R. This method requires a template life table from which it assesses the expected attrition rate. We used data from consecutive 2-monthly COVID-19 cohorts around the emergence of the variant of interest as templates (e.g. for the delta variant, we used as a template the comparison between those diagnosed with COVID-19 between May 20 and July 19, 2021 to those diagnosed between March 20 and May 19, 2021).

Using this method, we calculated index minimum sample sizes to achieve a power of 90% to detect a HR of 1.1 with an alpha of 0.01. We then inflated these index sample sizes to account for patients lost after matching (which we calculated to be at most 10%, when comparing consecutive 2-monthly cohorts of patients diagnosed with COVID-19) and for the effective prevalence of the variant of interest during the time windows of interest.

For instance, to define the sample size required for the delta variants, we estimated that the index minimum sample size had to be at least 20,700 per cohort. Inflating this to account for patients lost during matching (i.e. dividing by 0.9) lead to a sample size of 23,000. To recruit 23,000 patients expected to have the delta variant, given that at every point, the prevalence of the delta variant during that time window will be at least 50%, recruiting 46,000 people will be enough. Note that this is likely more people than is necessary because the prevalence of the variant of interest during the time windows selected were often much higher than 50% (see Fig. 3A).

Risk horizon and time to equal incidence

As described in the manuscript, a risk horizon was defined for each outcome as the time at which the time-varying HR reached 1 and a 'time to equal incidence' was defined as the time at which the cumulative incidence is equal between the two cohorts.

Outcomes can fall into different categories. If a risk horizon is never reached, then the number of *new* diagnoses being made continues to be higher after two years in the COVID-19 cohort compared to the other cohort. If a risk horizon is reached within two years but a time to equal incidence is not, then there comes a time when the number of new diagnoses being made is the same in the two cohorts, but the *total* number of diagnoses made since the index infection continues to be higher in the COVID-19 cohort. Finally, if a time to equal incidence is reached within two years, then the total number of diagnoses made in the control cohort has caught up with that in the COVID-19 cohort so that the total number of diagnoses made since the index infection is equal in the two cohorts.

A note on multiple comparisons

There are three reasons why we did not correct for multiple comparisons in our primary analyses (though they are reported in the Appendix):

1) We aimed to map the neurological and psychiatric sequelae of COVID-19 in as comprehensive a way as possible. Corrections for multiple comparisons disincentivises the inclusion of outcomes expected to be null such as parkinsonism in our case (as we had found no evidence of an increased risk of parkinsonism following COVID-19 in our previous study[5]). This is sometimes referred to as "penalty for peeking".[21]

2) Conversely, there are some outcomes which we already knew to be significantly associated with COVID-19. The current study aimed to refine their risk (by narrowing the confidence intervals), stratify it by subgroups, and map their trajectories, rather than to test whether their risk was significantly different from 1. Because of these outcomes, the "universal null hypothesis" (that all associations are due to chance) is already known to be false, which violates an assumption behind procedures to correct for multiple comparisons.[21]

3) Specifically for the analysis of variants, statistical power calculation was achieved by setting alpha to 0.05 for individual outcomes. If that threshold was lowered (to account for multiple comparisons), the time windows used to define likely exposure to variants would need to be broader which might increase the risk of bias which itself increases the risk of Type 1 errors. In other words, correcting for multiple comparisons would have traded a possible risk of Type 1 error caused by our observing multiple outcomes, for a likely risk of Type 1 error caused by actual bias. We do not believe this would have been desirable.

Assessing caveats in the analysis of variants

One possible caveat in the analysis of variant is that contextual factors (and notably disruption to health systems) might affect the rates at which psychiatric and neurological diagnoses are made. However, the follow-up time windows for patients diagnosed just after vs. just before the emergence of a variant largely overlapped (Table S19, p. 46 of this appendix) since the exposure time windows were close to each other. Moreover, we found little evidence of changes in the rates of neurological and psychiatric diagnoses between the different time windows in the general population of the TriNetX network (appendix p. 46). Only a small decrease in the number of diagnoses was observed after the emergence of the delta variant. The increased risk of neurological

and psychiatric diagnoses in patients with COVID-19 diagnosed after the emergence of the delta variant therefore occurred against a backdrop of a small decrease rate in the general population.

Another possible caveat is that milder variants, like omicron, might lead to fewer cases being formally diagnosed, so that those who receive a diagnosis represents a more severe subset of the cohort of interest. However, the biweekly incidence of confirmed COVID-19 diagnoses within the TriNetX US Collaborative Network between November 1, 2021 and March 6, 2022 (covering the transition from a delta dominance to an omicron dominance) was found to closely track the incidence of cases reported by the Centers for Disease Control and Prevention (https://covid.cdc.gov; r=0.97, p<0.0001). This suggests that no substantial change in the proportion of cases receiving a formal diagnosis occurred after the emergence of omicron.

References

1 Taquet M, Dercon Q, Luciano S, Geddes JR, Husain M, Harrison PJ. Incidence, co-occurrence, and evolution of long-COVID features: A 6-month retrospective cohort study of 273,618 survivors of COVID-19. *PLoS Med* 2021; **18**: e1003773.

2 Casey JA, Schwartz BS, Stewart WF, Adler NE. Using Electronic Health Records for Population Health Research: A Review of Methods and Applications. *Annu Rev Public Health* 2016; **37**: 61–81.

3 Cowie MR, Blomster JI, Curtis LH, *et al.* Electronic health records to facilitate clinical research. *Clin Res Cardiol* 2017; **106**: 1–9.

4 Jetley G, Zhang H. Electronic health records in IS research: Quality issues, essential thresholds and remedial actions. *Decis Support Syst* 2019; **126**: 113137.

5 Taquet M, Geddes JR, Husain M, Luciano S, Harrison PJ. 6-month neurological and psychiatric outcomes in 236 379 survivors of COVID-19: a retrospective cohort study using electronic health records. *Lancet Psychiatry* 2021; **8**: 416–27.

6 Taquet M, Dercon Q, Harrison PJ. Six-month sequelae of post-vaccination SARS-CoV-2 infection: A retrospective cohort study of 10,024 breakthrough infections. *Brain Behav Immun* 2022; **103**: 154–62.

7 Lu L, Xiong W, Liu D, *et al.* New onset acute symptomatic seizure and risk factors in coronavirus disease 2019: A retrospective multicenter study. *Epilepsia* 2020; **61**: e49–53.

8 Pellinen J, Carroll E, Friedman D, *et al.* Continuous EEG findings in patients with COVID-19 infection admitted to a New York academic hospital system. *Epilepsia* 2020; **61**: 2097–105.

9 Taquet M, Devinsky O, Cross H, Harrison PJ, Sen A. Incidence of epilepsy and seizures over the first 6 months after a COVID-19 diagnosis: a retrospective cohort study. *Neurology (in press)*.

10 de Lusignan S, Dorward J, Correa A, *et al.* Risk factors for SARS-CoV-2 among patients in the Oxford Royal College of General Practitioners Research and Surveillance Centre primary care network: a cross-sectional study. *Lancet Infect Dis* 2020; published online May 15. DOI:10.1016/S1473-3099(20)30371-6.

11 Zhang J-J, Dong X, Cao Y-Y, *et al.* Clinical characteristics of 140 patients infected with SARS-CoV-2 in Wuhan, China. *Allergy* 2020. https://onlinelibrary.wiley.com/doi/abs/10.1111/all.14238.

12 Chen N, Zhou M, Dong X, *et al.* Epidemiological and clinical characteristics of 99 cases of 2019 novel coronavirus pneumonia in Wuhan, China: a descriptive study. *Lancet* 2020; **395**: 507–13.

13 Taquet M, Luciano S, Geddes JR, Harrison PJ. Bidirectional associations between COVID-19 and psychiatric disorder: retrospective cohort studies of 62 354 COVID-19 cases in the USA. *Lancet Psychiatry* 2021; **8**: 130–40.

14 Williamson EJ, Walker AJ, Bhaskaran K, *et al.* Factors associated with COVID-19-related death using OpenSAFELY. *Nature* 2020; **584**: 430–6.

15 De Picker LJ, Leboyer M, Geddes JR, Morrens M, Harrison PJ, Taquet M. Association between serum lithium level and incidence of COVID-19 infection. *Br J Psychiatry* 2022; : 1–3.

16 Lenze EJ, Mattar C, Zorumski CF, *et al.* Fluvoxamine vs Placebo and Clinical Deterioration in Outpatients With Symptomatic COVID-19: A Randomized Clinical Trial. *JAMA* 2020; **324**: 2292–300.

17 Vai B, Mazza MG, Colli CD, *et al.* Mental disorders and risk of COVID-19-related mortality, hospitalisation, and intensive care unit admission: a systematic review and meta-analysis. The Lancet Psychiatry. 2021; **8**: 797–812.

18 Royston P, Parmar MKB. Flexible parametric proportional-hazards and proportional-odds models for censored survival data, with application to prognostic modelling and estimation of treatment effects. Statistics in Medicine. 2002; **21**: 2175–97.

19 Liu X-R, Pawitan Y, Clements M. Parametric and penalized generalized survival models. *Stat Methods Med Res* 2018; **27**: 1531–46.

20 Freedman LS. Tables of the number of patients required in clinical trials using the logrank test. *Stat Med* 1982; **1**: 121–9.

21 Rothman KJ. No adjustments are needed for multiple comparisons. *Epidemiology* 1990; **1**: 43–6.

Supplementary figures

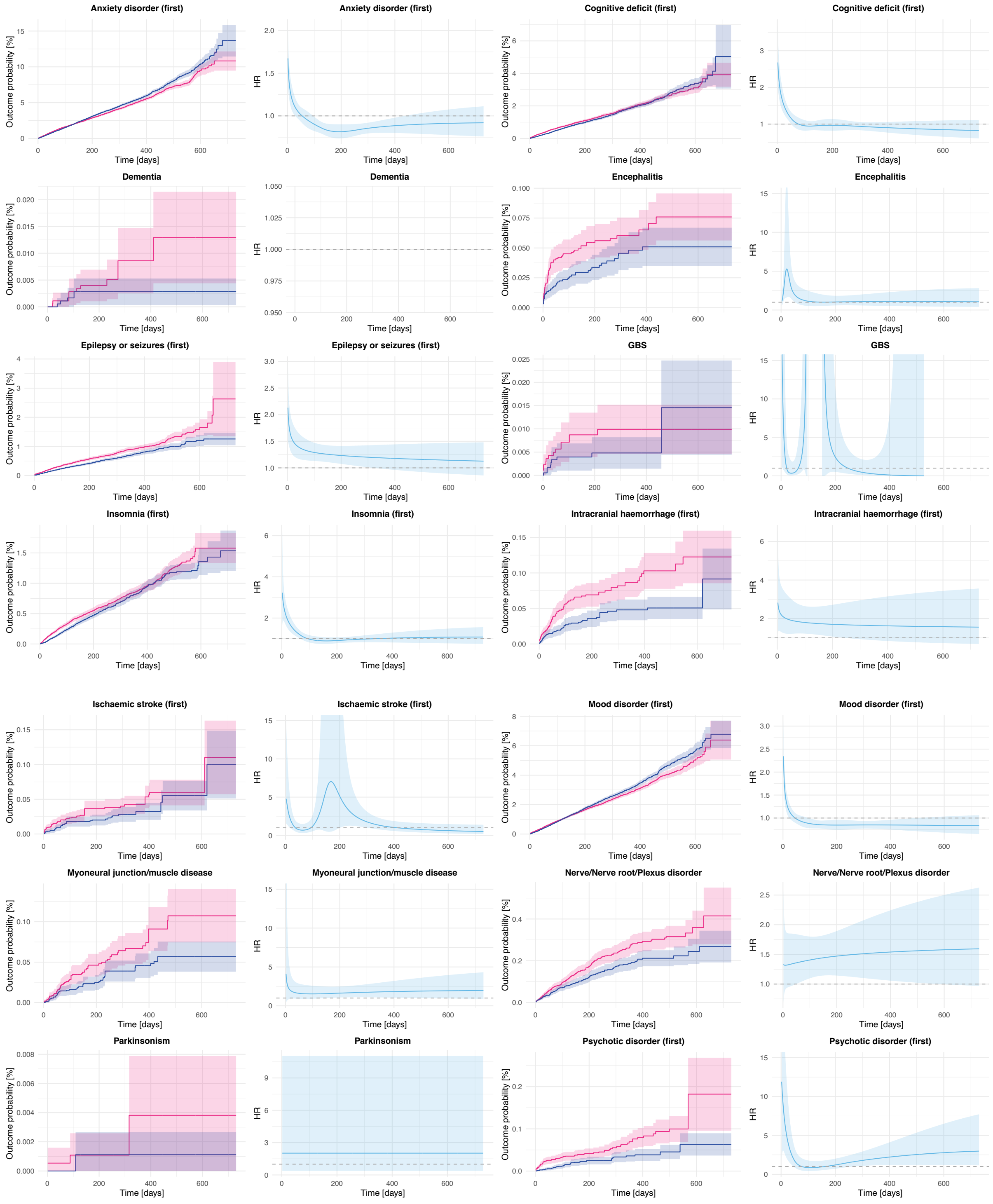

Fig. S1 – Kaplan-Meier curves (left panel for each outcome; red for COVID-19 and blue for other respiratory tract infection) and time-varying HRs (right panel for each outcome) for the different outcomes in children. Shaded areas around curves represent 95% CI.

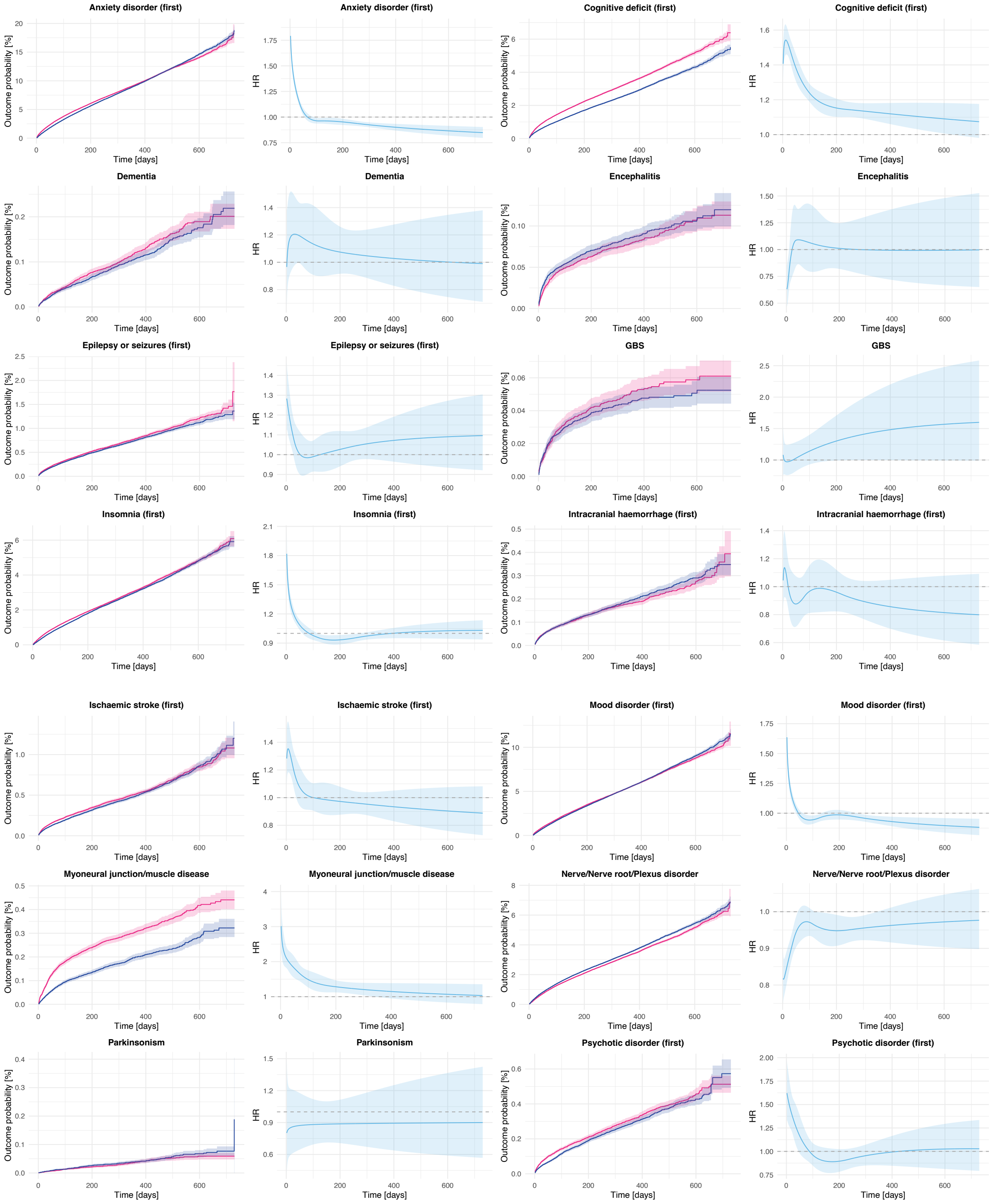

Fig. S2 – Kaplan-Meier curves (left panel for each outcome; red for COVID-19 and blue for other respiratory tract infection) and time-varying HRs (right panel for each outcome) for the different outcomes in adults. Shaded areas around curves represent 95% CI.

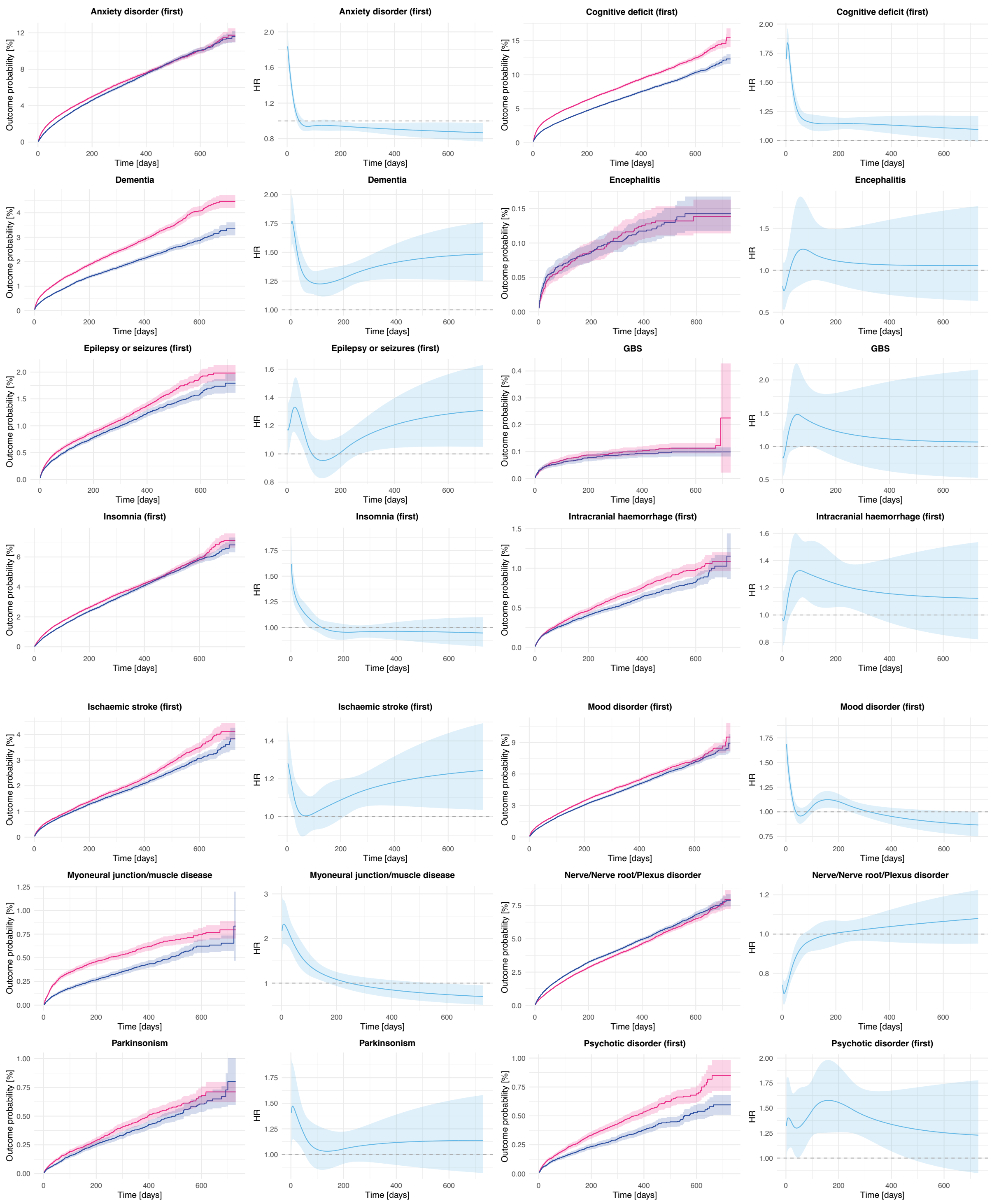

Fig. S3 – Kaplan-Meier curves (left panel for each outcome; red for COVID-19 and blue for other respiratory tract infection) and time-varying HRs (right panel for each outcome) for the different outcomes in older adults. Shaded areas around curves represent 95% CI.

Supplementary tables

Table S1 - All baseline characteristics used in the propensity score matching as well as the distribution of index dates used for stratification. A baseline characteristic with a standardized mean difference (SMD) less than 0.1 is considered well matched.

	COVID (Unmatched)	COVID (Matched)	Other Resp. (Matched)	SMD
**Number**	1487712	1284437	1284437	
**DEMOGRAPHICS**				
**Age; mean (SD)**	44.0 (21.6)	42.5 (21.9)	42.6 (22.1)	0.008
**Sex; n (%)**				
**Female**	822711 (55.3)	741806 (57.8)	741696 (57.7)	0.0002
**Male**	664460 (44.7)	542192 (42.2)	542305 (42.2)	0.0002
**Other**	663 (0.0)	576 (0.0)	573 (0.0)	0.0001
**Race; n (%)**				
**White**	832557 (56.0)	745846 (58.1)	745452 (58.0)	0.0006
**Black or African American**	250764 (16.9)	203616 (15.9)	203086 (15.8)	0.001
**Asian**	36464 (2.5)	29864 (2.3)	30166 (2.3)	0.002
**American Indian or Alaska Native**	5685 (0.4)	4780 (0.4)	4671 (0.4)	0.001
**Native Hawaiian or Other Pacific Islander**	2431 (0.2)	1835 (0.1)	1791 (0.1)	0.0009
**Unknown**	359849 (24.2)	298536 (23.2)	299316 (23.3)	0.001
**Ethnicity; n (%)**				
**Hispanic or Latino**	189622 (12.7)	146593 (11.4)	146910 (11.4)	0.0008
**Not Hispanic or Latino**	947086 (63.7)	834868 (65.0)	830486 (64.7)	0.007
**Unknown**	351004 (23.6)	302976 (23.6)	307041 (23.9)	0.007
**Problems related to housing and economic circumstances; n (%)**	28196 (1.9)	26567 (2.1)	27106 (2.1)	0.003
**COMORBIDITIES**				
**Overweight and obesity; n (%)**	320520 (21.5)	267574 (20.8)	265006 (20.6)	0.005
**Hypertensive disease; n (%)**				
**Hypertensive diseases**	469519 (31.6)	392616 (30.6)	388894 (30.3)	0.006
**Hypertensive diseases (deprecated 2018)**	468933 (31.5)	392171 (30.5)	388456 (30.2)	0.006
**Diabetes mellitus; n (%)**				
**Type 1 diabetes mellitus**	29614 (2.0)	24230 (1.9)	24008 (1.9)	0.001
**Type 2 diabetes mellitus**	238094 (16.0)	186867 (14.5)	186048 (14.5)	0.002
**Chronic lower respiratory diseases; n (%)**				
**Asthma**	190561 (12.8)	179381 (14.0)	180792 (14.1)	0.003
**Bronchiectasis**	8511 (0.6)	8310 (0.6)	9470 (0.7)	0.01
**Bronchitis, not specified as acute or chronic**	85358 (5.7)	81692 (6.4)	84159 (6.6)	0.008
**Emphysema**	26014 (1.7)	24652 (1.9)	26145 (2.0)	0.008
**Other chronic obstructive pulmonary disease**	77183 (5.2)	73255 (5.7)	75799 (5.9)	0.008
**Simple and mucopurulent chronic bronchitis**	6348 (0.4)	6211 (0.5)	7065 (0.6)	0.009
**Unspecified chronic bronchitis**	8000 (0.5)	7814 (0.6)	8963 (0.7)	0.01
**Nicotine dependence; n (%)**	153651 (10.3)	143014 (11.1)	144603 (11.3)	0.004

**Psychiatric comorbidities; n (%)**				
**Anxiety disorders**	337877 (22.7)	315075 (24.5)	314335 (24.5)	0.001
**Substance misuse**	208249 (14.0)	191590 (14.9)	192580 (15.0)	0.002
**Mood disorders**	260720 (17.5)	240583 (18.7)	240747 (18.7)	0.0003
**Psychotic disorders**	25134 (1.7)	21915 (1.7)	22037 (1.7)	0.0007
**Heart disease; n (%)**				
**Ischemic heart diseases**	149630 (10.1)	127137 (9.9)	127219 (9.9)	0.0002
**Other forms of heart disease**	285499 (19.2)	245389 (19.1)	244229 (19.0)	0.002
**Chronic kidney diseases; n (%)**				
**Chronic kidney disease (CKD)**	112982 (7.6)	94726 (7.4)	94474 (7.4)	0.0008
**Hypertensive chronic kidney disease**	57297 (3.9)	45066 (3.5)	44761 (3.5)	0.001
**Chronic liver diseases; n (%)**				
**Alcoholic liver disease**	6954 (0.5)	6183 (0.5)	6242 (0.5)	0.0007
**Chronic hepatitis, not elsewhere classified**	1928 (0.1)	1814 (0.1)	1944 (0.2)	0.003
**Chronic passive congestion of liver**	7817 (0.5)	7064 (0.5)	7173 (0.6)	0.001
**Fatty (change of) liver, not elsewhere classified**	57873 (3.9)	48691 (3.8)	48657 (3.8)	0.0001
**Fibrosis and cirrhosis of liver**	17609 (1.2)	15262 (1.2)	15478 (1.2)	0.002
**Hepatic failure, not elsewhere classified**	10052 (0.7)	8771 (0.7)	8963 (0.7)	0.002
**Other specified diseases of liver**	32472 (2.2)	28587 (2.2)	28763 (2.2)	0.0009
**Portal hypertension**	7062 (0.5)	6056 (0.5)	6078 (0.5)	0.0002
**Cerebral infarction; n (%)**	47379 (3.2)	40211 (3.1)	40555 (3.2)	0.002
**Dementia; n (%)**				
**Alzheimer's disease**	7601 (0.5)	5342 (0.4)	5273 (0.4)	0.0008
**Dementia in other diseases classified elsewhere**	9929 (0.7)	7148 (0.6)	7074 (0.6)	0.0008
**Dementia with Lewy bodies**	863 (0.1)	679 (0.1)	678 (0.1)	0.00003
**Frontotemporal dementia**	538 (0.0)	398 (0.0)	403 (0.0)	0.0002
**Unspecified dementia**	22176 (1.5)	16734 (1.3)	16712 (1.3)	0.0002
**Vascular dementia**	5287 (0.4)	4004 (0.3)	3988 (0.3)	0.0002
**Neoplasms; n (%)**				
**Malignant neoplasms of lymphoid, hematopoietic and related tissue**	18789 (1.3)	17378 (1.4)	17268 (1.3)	0.0007
**Neoplasms (benign or malignant)**	302578 (20.3)	275015 (21.4)	274985 (21.4)	0.00006
**Organ transplant; n (%)**				
**Liver Transplantation Procedures**	841 (0.1)	710 (0.1)	663 (0.1)	0.002
**Renal Transplantation Procedures**	3455 (0.2)	2139 (0.2)	2055 (0.2)	0.002
**Psoriasis; n (%)**	19385 (1.3)	17667 (1.4)	17689 (1.4)	0.0001
**Rheumatoid arthritis; n (%)**				
**Other rheumatoid arthritis**	25897 (1.7)	23507 (1.8)	23786 (1.9)	0.002
**Rheumatoid arthritis with rheumatoid factor**	7017 (0.5)	6287 (0.5)	6431 (0.5)	0.002
**Systemic lupus erythematosus (SLE); n (%)**	9552 (0.6)	8506 (0.7)	8599 (0.7)	0.0009
**Disorders involving the immune mechanism; n (%)**	42718 (2.9)	37118 (2.9)	37444 (2.9)	0.002
**MEDICATIONS**				
**COVID-19 vaccine; n (%)**				
**Pfizer**	21765 (1.5)	20293 (1.6)	20953 (1.6)	0.004

**Moderna**	3209 (0.2)	3061 (0.2)	3273 (0.3)	0.003
**Janssen**	598 (0.0)	583 (0.0)	597 (0.0)	0.0005
**Not specified**	35278 (2.4)	33156 (2.6)	33914 (2.6)	0.004
**Antidepressants; n (%)**				
**Any**	342395 (23.0)	318657 (24.8)	318406 (24.8)	0.0005
**Fluvoxamine**	2132 (0.1)	1916 (0.1)	1885 (0.1)	0.0006
**Lithium; n (%)**	4517 (0.3)	4214 (0.3)	4292 (0.3)	0.001
**Antipsychotics; n (%)**				
**Any**	110197 (7.4)	97337 (7.6)	97186 (7.6)	0.0004
**Clozapine**	917 (0.1)	761 (0.1)	736 (0.1)	0.0008
**INDEX EVENT; n (%)**				
**Feb-Mar 2020**	257181 (17.3)	173856 (13.5)	173856 (13.5)	0
**Apr-May 2020**	2481 (0.2)	2444 (0.2)	2444 (0.2)	0
**Jun-Jul 2020**	57804 (3.9)	55638 (4.3)	55638 (4.3)	0
**Aug-Sep 2020**	82570 (5.6)	74737 (5.8)	74737 (5.8)	0
**Oct-Nov 2020**	84055 (5.6)	81329 (6.3)	81329 (6.3)	0
**Dec-Jan 2021**	139847 (9.4)	128925 (10)	128925 (10)	0
**Feb-Mar 2021**	294240 (19.8)	248429 (19.3)	248429 (19.3)	0
**Apr-May 2021**	117173 (7.9)	102756 (8)	102756 (8)	0
**Jun-Jul 2021**	83969 (5.6)	80651 (6.3)	80651 (6.3)	0
**Aug-Sep 2021**	41793 (2.8)	41776 (3.3)	41776 (3.3)	0
**Oct-Nov 2021**	187278 (12.6)	160516 (12.5)	160516 (12.5)	0
**Dec-Jan 2022**	139321 (9.4)	133380 (10.4)	133380 (10.4)	0

Table S2 - All baseline characteristics for the cohorts of children. A baseline characteristic with a standardized mean difference (SMD) less than 0.1 is considered well matched.

	COVID (Unmatched)	COVID (Matched)	Other Resp. (Matched)	SMD
**Number**	186448	185748	185748	
**DEMOGRAPHICS**				
**Age; mean (SD)**	9.0 (5.7)	8.9 (5.6)	8.9 (5.7)	0.002
**Sex; n (%)**				
**Female**	91998 (49.3)	91694 (49.4)	91951 (49.5)	0.003
**Male**	94396 (50.6)	94007 (50.6)	93763 (50.5)	0.003
**Other**	106 (0.1)	104 (0.1)	100 (0.1)	0.0009
**Race; n (%)**				
**White**	93995 (50.4)	93906 (50.6)	93920 (50.6)	0.0002
**Black or African American**	35921 (19.3)	35524 (19.1)	35171 (18.9)	0.005
**Asian**	4722 (2.5)	4704 (2.5)	4863 (2.6)	0.005
**American Indian or Alaska Native**	703 (0.4)	702 (0.4)	677 (0.4)	0.002
**Native Hawaiian or Other Pacific Islander**	304 (0.2)	302 (0.2)	308 (0.2)	0.0008
**Unknown**	50833 (27.3)	50640 (27.3)	50843 (27.4)	0.002
**Ethnicity; n (%)**				
**Hispanic or Latino**	32253 (17.3)	32076 (17.3)	31744 (17.1)	0.005
**Not Hispanic or Latino**	112663 (60.4)	112321 (60.5)	112023 (60.3)	0.003
**Unknown**	41532 (22.3)	41351 (22.3)	41981 (22.6)	0.008
**Problems related to housing and economic circumstances; n (%)**	1707 (0.9)	1695 (0.9)	1613 (0.9)	0.005
**COMORBIDITIES**				
**Overweight and obesity; n (%)**	13363 (7.2)	13187 (7.1)	12672 (6.8)	0.01
**Hypertensive disease; n (%)**				
**Hypertensive diseases**	4045 (2.2)	3894 (2.1)	3377 (1.8)	0.02
**Hypertensive diseases (deprecated 2018)**	4039 (2.2)	3889 (2.1)	3372 (1.8)	0.02
**Diabetes mellitus; n (%)**				
**Type 1 diabetes mellitus**	1206 (0.6)	1147 (0.6)	1008 (0.5)	0.01
**Type 2 diabetes mellitus**	3077 (1.7)	2959 (1.6)	2552 (1.4)	0.02
**Chronic lower respiratory diseases; n (%)**				
**Asthma**	27093 (14.5)	26988 (14.5)	26165 (14.1)	0.01
**Bronchiectasis**	212 (0.1)	211 (0.1)	200 (0.1)	0.002
**Bronchitis, not specified as acute or chronic**	5299 (2.8)	5287 (2.8)	5086 (2.7)	0.007
**Emphysema**	152 (0.1)	145 (0.1)	140 (0.1)	0.001
**Other chronic obstructive pulmonary disease**	468 (0.3)	461 (0.2)	404 (0.2)	0.006
**Simple and mucopurulent chronic bronchitis**	144 (0.1)	142 (0.1)	116 (0.1)	0.005
**Unspecified chronic bronchitis**	149 (0.1)	149 (0.1)	128 (0.1)	0.004
**Nicotine dependence; n (%)**	1555 (0.8)	1508 (0.8)	1328 (0.7)	0.01
**Psychiatric comorbidities; n (%)**				
**Anxiety disorders**	17676 (9.5)	17542 (9.4)	16312 (8.8)	0.02

**Substance misuse**	3321 (1.8)	3228 (1.7)	2843 (1.5)	0.02
**Mood disorders**	9067 (4.9)	8957 (4.8)	8265 (4.4)	0.02
**Psychotic disorders**	689 (0.4)	660 (0.4)	574 (0.3)	0.008
**Heart disease; n (%)**				
**Ischemic heart diseases**	721 (0.4)	705 (0.4)	617 (0.3)	0.008
**Other forms of heart disease**	7954 (4.3)	7799 (4.2)	7390 (4.0)	0.01
**Chronic kidney diseases; n (%)**				
**Chronic kidney disease (CKD)**	1111 (0.6)	1076 (0.6)	947 (0.5)	0.009
**Hypertensive chronic kidney disease**	281 (0.2)	257 (0.1)	224 (0.1)	0.005
**Chronic liver diseases; n (%)**				
**Alcoholic liver disease**	30 (0.0)	20 (0.0)	30 (0.0)	0.005
**Chronic hepatitis, not elsewhere classified**	102 (0.1)	81 (0.0)	90 (0.0)	0.002
**Chronic passive congestion of liver**	127 (0.1)	125 (0.1)	129 (0.1)	0.0008
**Fatty (change of) liver, not elsewhere classified**	738 (0.4)	687 (0.4)	592 (0.3)	0.009
**Fibrosis and cirrhosis of liver**	187 (0.1)	175 (0.1)	156 (0.1)	0.003
**Hepatic failure, not elsewhere classified**	184 (0.1)	175 (0.1)	156 (0.1)	0.003
**Other specified diseases of liver**	455 (0.2)	427 (0.2)	380 (0.2)	0.005
**Portal hypertension**	118 (0.1)	116 (0.1)	108 (0.1)	0.002
**Cerebral infarction; n (%)**	1433 (0.8)	1363 (0.7)	1226 (0.7)	0.009
**Dementia; n (%)**				
**Alzheimer's disease**	30 (0.0)	0 (0.0)	10 (0.0)	0.01
**Dementia in other diseases classified elsewhere**	80 (0.0)	60 (0.0)	50 (0.0)	0.003
**Dementia with Lewy bodies**	20 (0.0)	20 (0.0)	10 (0.0)	0.006
**Frontotemporal dementia**	20 (0.0)	0 (0.0)	0 (0.0)	
**Unspecified dementia**	100 (0.1)	80 (0.0)	80 (0.0)	0
**Vascular dementia**	50 (0.0)	30 (0.0)	40 (0.0)	0.004
**Neoplasms; n (%)**				
**Malignant neoplasms of lymphoid, hematopoietic and related tissue**	731 (0.4)	694 (0.4)	624 (0.3)	0.006
**Neoplasms (benign or malignant)**	9116 (4.9)	9025 (4.9)	8718 (4.7)	0.008
**Organ transplant; n (%)**				
**Liver Transplantation Procedures**	110 (0.1)	110 (0.1)	90 (0.0)	0.005
**Renal Transplantation Procedures**	117 (0.1)	114 (0.1)	82 (0.0)	0.008
**Psoriasis; n (%)**	458 (0.2)	455 (0.2)	403 (0.2)	0.006
**Rheumatoid arthritis; n (%)**				
**Other rheumatoid arthritis**	203 (0.1)	195 (0.1)	170 (0.1)	0.004
**Rheumatoid arthritis with rheumatoid factor**	50 (0.0)	10 (0.0)	10 (0.0)	0
**Systemic lupus erythematosus (SLE); n (%)**	189 (0.1)	172 (0.1)	158 (0.1)	0.003
**Disorders involving the immune mechanism; n (%)**	2558 (1.4)	2469 (1.3)	2266 (1.2)	0.01
**MEDICATIONS**				
**COVID-19 vaccine; n (%)**				
**Pfizer**	1198 (0.6)	1190 (0.6)	1119 (0.6)	0.005
**Moderna**	10 (0.0)	10 (0.0)	0 (0.0)	0.01
**Janssen**	0 (0.0)	0 (0.0)	0 (0.0)	

**Not specified**	1890 (1.0)	1869 (1.0)	1774 (1.0)	0.005
**Antidepressants; n (%)**				
**Any**	8960 (4.8)	8895 (4.8)	8174 (4.4)	0.02
**Fluvoxamine**	114 (0.1)	113 (0.1)	113 (0.1)	0
**Lithium; n (%)**	132 (0.1)	132 (0.1)	142 (0.1)	0.002
**Antipsychotics; n (%)**				
**Any**	2974 (1.6)	2918 (1.6)	2590 (1.4)	0.01
**Clozapine**	40 (0.0)	30 (0.0)	20 (0.0)	0.005
INDEX EVENT; n (%)				
**Feb-Mar 2020**	21929 (11.8)	21670 (11.7)	21670 (11.7)	0
**Apr-May 2020**	82 (0)	81 (0)	81 (0)	0
**Jun-Jul 2020**	1865 (1)	1853 (1)	1853 (1)	0
**Aug-Sep 2020**	5634 (3)	5608 (3)	5608 (3)	0
**Oct-Nov 2020**	6686 (3.6)	6672 (3.6)	6672 (3.6)	0
**Dec-Jan 2021**	13919 (7.5)	13903 (7.5)	13903 (7.5)	0
**Feb-Mar 2021**	56586 (30.3)	56298 (30.3)	56298 (30.3)	0
**Apr-May 2021**	11886 (6.4)	11876 (6.4)	11876 (6.4)	0
**Jun-Jul 2021**	10592 (5.7)	10584 (5.7)	10584 (5.7)	0
**Aug-Sep 2021**	4867 (2.6)	4862 (2.6)	4862 (2.6)	0
**Oct-Nov 2021**	29427 (15.8)	29371 (15.8)	29371 (15.8)	0
**Dec-Jan 2022**	22975 (12.3)	22970 (12.4)	22970 (12.4)	0

Table S3 - All baseline characteristics for the cohorts of adults. A baseline characteristic with a standardized mean difference (SMD) less than 0.1 is considered well matched.

	COVID (Unmatched)	COVID (Matched)	Other Resp. (Matched)	SMD
**Number**	1010151	856588	856588	
**DEMOGRAPHICS**				
**Age; mean (SD)**	41.8 (13.3)	40.9 (13.3)	41.1 (13.9)	0.02
**Sex; n (%)**				
**Female**	578064 (57.2)	517461 (60.4)	517887 (60.5)	0.001
**Male**	431647 (42.7)	338772 (39.5)	338333 (39.5)	0.001
**Other**	447 (0.0)	362 (0.0)	373 (0.0)	0.0006
**Race; n (%)**				
**White**	546299 (54.1)	487663 (56.9)	487889 (57.0)	0.0005
**Black or African American**	178469 (17.7)	140382 (16.4)	139805 (16.3)	0.002
**Asian**	25228 (2.5)	20316 (2.4)	20475 (2.4)	0.001
**American Indian or Alaska Native**	4143 (0.4)	3440 (0.4)	3363 (0.4)	0.001
**Native Hawaiian or Other Pacific Islander**	1825 (0.2)	1337 (0.2)	1296 (0.2)	0.001
**Unknown**	254188 (25.2)	203451 (23.8)	203763 (23.8)	0.0009
**Ethnicity; n (%)**				
**Hispanic or Latino**	135135 (13.4)	99521 (11.6)	99838 (11.7)	0.001
**Not Hispanic or Latino**	634372 (62.8)	552984 (64.6)	549319 (64.1)	0.009
**Unknown**	240644 (23.8)	204083 (23.8)	207431 (24.2)	0.009
**Problems related to housing and economic circumstances; n (%)**	19791 (2.0)	18631 (2.2)	19145 (2.2)	0.004
**COMORBIDITIES**				
**Overweight and obesity; n (%)**	233020 (23.1)	191323 (22.3)	189569 (22.1)	0.005
**Hypertensive disease; n (%)**				
**Hypertensive diseases**	271672 (26.9)	222226 (25.9)	221224 (25.8)	0.003
**Hypertensive diseases (deprecated 2018)**	271284 (26.9)	221936 (25.9)	220930 (25.8)	0.003
**Diabetes mellitus; n (%)**				
**Type 1 diabetes mellitus**	18263 (1.8)	14528 (1.7)	14472 (1.7)	0.0005
**Type 2 diabetes mellitus**	138707 (13.7)	105308 (12.3)	105154 (12.3)	0.0005
**Chronic lower respiratory diseases; n (%)**				
**Asthma**	129533 (12.8)	120157 (14.0)	121734 (14.2)	0.005
**Bronchiectasis**	3743 (0.4)	3596 (0.4)	4077 (0.5)	0.008
**Bronchitis, not specified as acute or chronic**	57745 (5.7)	54889 (6.4)	56739 (6.6)	0.009
**Emphysema**	9826 (1.0)	9292 (1.1)	10229 (1.2)	0.01
**Other chronic obstructive pulmonary disease**	31963 (3.2)	30599 (3.6)	32697 (3.8)	0.01
**Simple and mucopurulent chronic bronchitis**	2703 (0.3)	2645 (0.3)	3110 (0.4)	0.009
**Unspecified chronic bronchitis**	3867 (0.4)	3760 (0.4)	4407 (0.5)	0.01
**Nicotine dependence; n (%)**	123653 (12.2)	114704 (13.4)	116233 (13.6)	0.005
**Psychiatric comorbidities; n (%)**				
**Anxiety disorders**	249192 (24.7)	231478 (27.0)	232505 (27.1)	0.003
**Substance misuse**	164160 (16.3)	150306 (17.5)	151422 (17.7)	0.003

**Mood disorders**	188532 (18.7)	174304 (20.3)	175627 (20.5)	0.004
**Psychotic disorders**	16888 (1.7)	15064 (1.8)	15215 (1.8)	0.001
**Heart disease; n (%)**				
**Ischemic heart diseases**	61754 (6.1)	50923 (5.9)	51438 (6.0)	0.003
**Other forms of heart disease**	152422 (15.1)	127744 (14.9)	128091 (15.0)	0.001
**Chronic kidney diseases; n (%)**				
**Chronic kidney disease (CKD)**	49273 (4.9)	39798 (4.6)	40179 (4.7)	0.002
**Hypertensive chronic kidney disease**	23838 (2.4)	17379 (2.0)	17378 (2.0)	0.000008
**Chronic liver diseases; n (%)**				
**Alcoholic liver disease**	5143 (0.5)	4577 (0.5)	4602 (0.5)	0.0004
**Chronic hepatitis, not elsewhere classified**	1185 (0.1)	1116 (0.1)	1206 (0.1)	0.003
**Chronic passive congestion of liver**	4650 (0.5)	4105 (0.5)	4102 (0.5)	0.00005
**Fatty (change of) liver, not elsewhere classified**	43258 (4.3)	35790 (4.2)	35830 (4.2)	0.0002
**Fibrosis and cirrhosis of liver**	10849 (1.1)	9322 (1.1)	9421 (1.1)	0.001
**Hepatic failure, not elsewhere classified**	6410 (0.6)	5564 (0.6)	5634 (0.7)	0.001
**Other specified diseases of liver**	20789 (2.1)	17867 (2.1)	17959 (2.1)	0.0008
**Portal hypertension**	4546 (0.5)	3833 (0.4)	3828 (0.4)	0.00009
**Cerebral infarction; n (%)**	22246 (2.2)	18615 (2.2)	18970 (2.2)	0.003
**Dementia; n (%)**				
**Alzheimer's disease**	407 (0.0)	338 (0.0)	315 (0.0)	0.001
**Dementia in other diseases classified elsewhere**	860 (0.1)	743 (0.1)	732 (0.1)	0.0004
**Dementia with Lewy bodies**	113 (0.0)	111 (0.0)	100 (0.0)	0.001
**Frontotemporal dementia**	129 (0.0)	118 (0.0)	116 (0.0)	0.0002
**Unspecified dementia**	2289 (0.2)	1938 (0.2)	1992 (0.2)	0.001
**Vascular dementia**	680 (0.1)	556 (0.1)	564 (0.1)	0.0004
**Neoplasms; n (%)**				
**Malignant neoplasms of lymphoid, hematopoietic and related tissue**	9102 (0.9)	8225 (1.0)	8309 (1.0)	0.001
**Neoplasms (benign or malignant)**	187557 (18.6)	167862 (19.6)	169715 (19.8)	0.005
**Organ transplant; n (%)**				
**Liver Transplantation Procedures**	479 (0.0)	393 (0.0)	371 (0.0)	0.001
**Renal Transplantation Procedures**	2581 (0.3)	1504 (0.2)	1439 (0.2)	0.002
**Psoriasis; n (%)**	13174 (1.3)	11886 (1.4)	11958 (1.4)	0.0007
**Rheumatoid arthritis; n (%)**				
**Other rheumatoid arthritis**	15032 (1.5)	13544 (1.6)	13714 (1.6)	0.002
**Rheumatoid arthritis with rheumatoid factor**	4082 (0.4)	3591 (0.4)	3644 (0.4)	0.001
**Systemic lupus erythematosus (SLE); n (%)**	7445 (0.7)	6537 (0.8)	6608 (0.8)	0.0009
**Disorders involving the immune mechanism; n (%)**	28058 (2.8)	23673 (2.8)	23997 (2.8)	0.002
**MEDICATIONS**				
**COVID-19 vaccine; n (%)**				
**Pfizer**	13748 (1.4)	12614 (1.5)	13182 (1.5)	0.005
**Moderna**	1785 (0.2)	1682 (0.2)	1790 (0.2)	0.003
**Janssen**	428 (0.0)	417 (0.0)	433 (0.1)	0.0008
**Not specified**	22868 (2.3)	21277 (2.5)	21929 (2.6)	0.005

**Antidepressants; n (%)**				
**Any**	245109 (24.3)	229566 (26.8)	230261 (26.9)	0.002
**Fluvoxamine**	1603 (0.2)	1467 (0.2)	1447 (0.2)	0.0006
**Lithium; n (%)**	3767 (0.4)	3557 (0.4)	3603 (0.4)	0.0008
**Antipsychotics; n (%)**				
**Any**	75598 (7.5)	67597 (7.9)	68076 (7.9)	0.002
**Clozapine**	647 (0.1)	559 (0.1)	529 (0.1)	0.001
**INDEX EVENT; n (%)**				
**Feb-Mar 2020**	173056 (17.1)	115814 (13.5)	115814 (13.5)	0
**Apr-May 2020**	1638 (0.2)	1630 (0.2)	1630 (0.2)	0
**Jun-Jul 2020**	40710 (4)	39537 (4.6)	39537 (4.6)	0
**Aug-Sep 2020**	62458 (6.2)	55586 (6.5)	55586 (6.5)	0
**Oct-Nov 2020**	62297 (6.2)	60178 (7)	60178 (7)	0
**Dec-Jan 2021**	98013 (9.7)	89648 (10.5)	89648 (10.5)	0
**Feb-Mar 2021**	190862 (18.9)	152656 (17.8)	152656 (17.8)	0
**Apr-May 2021**	78402 (7.8)	67985 (7.9)	67985 (7.9)	0
**Jun-Jul 2021**	59616 (5.9)	56516 (6.6)	56516 (6.6)	0
**Aug-Sep 2021**	29069 (2.9)	29065 (3.4)	29065 (3.4)	0
**Oct-Nov 2021**	125363 (12.4)	103109 (12)	103109 (12)	0
**Dec-Jan 2022**	88667 (8.8)	84864 (9.9)	84864 (9.9)	0

Table S4 - All baseline characteristics for the cohorts of older adults. A baseline characteristic with a standardized mean difference (SMD) less than 0.1 is considered well matched.

	COVID (Unmatched)	COVID (Matched)	Other Resp. (Matched)	SMD
**Number**	291113	242101	242101	
**DEMOGRAPHICS**				
**Age; mean (SD)**	73.9 (6.6)	73.8 (6.6)	73.8 (6.6)	0.004
**Sex; n (%)**				
**Female**	152649 (52.4)	132651 (54.8)	131858 (54.5)	0.007
**Male**	138417 (47.5)	109413 (45.2)	110209 (45.5)	0.007
**Other**	110 (0.0)	110 (0.0)	100 (0.0)	0.002
**Race; n (%)**				
**White**	192263 (66.0)	164277 (67.9)	163643 (67.6)	0.006
**Black or African American**	36374 (12.5)	27710 (11.4)	28110 (11.6)	0.005
**Asian**	6514 (2.2)	4844 (2.0)	4828 (2.0)	0.0005
**American Indian or Alaska Native**	839 (0.3)	638 (0.3)	631 (0.3)	0.0006
**Native Hawaiian or Other Pacific Islander**	302 (0.1)	196 (0.1)	187 (0.1)	0.001
**Unknown**	54828 (18.8)	44445 (18.4)	44710 (18.5)	0.003
**Ethnicity; n (%)**				
**Hispanic or Latino**	22234 (7.6)	14996 (6.2)	15328 (6.3)	0.006
**Not Hispanic or Latino**	200051 (68.7)	169563 (70.0)	169144 (69.9)	0.004
**Unknown**	68828 (23.6)	57542 (23.8)	57629 (23.8)	0.0008
**Problems related to housing and economic circumstances; n (%)**	6698 (2.3)	6241 (2.6)	6348 (2.6)	0.003
**COMORBIDITIES**				
**Overweight and obesity; n (%)**	74137 (25.5)	63064 (26.0)	62765 (25.9)	0.003
**Hypertensive disease; n (%)**				
**Hypertensive diseases**	193802 (66.6)	166496 (68.8)	164293 (67.9)	0.02
**Hypertensive diseases (deprecated 2018)**	193610 (66.5)	166346 (68.7)	164154 (67.8)	0.02
**Diabetes mellitus; n (%)**				
**Type 1 diabetes mellitus**	10145 (3.5)	8555 (3.5)	8528 (3.5)	0.0006
**Type 2 diabetes mellitus**	96310 (33.1)	78600 (32.5)	78342 (32.4)	0.002
**Chronic lower respiratory diseases; n (%)**				
**Asthma**	33935 (11.7)	32236 (13.3)	32893 (13.6)	0.008
**Bronchiectasis**	4556 (1.6)	4503 (1.9)	5193 (2.1)	0.02
**Bronchitis, not specified as acute or chronic**	22314 (7.7)	21516 (8.9)	22334 (9.2)	0.01
**Emphysema**	16036 (5.5)	15215 (6.3)	15776 (6.5)	0.009
**Other chronic obstructive pulmonary disease**	44752 (15.4)	42195 (17.4)	42698 (17.6)	0.005
**Simple and mucopurulent chronic bronchitis**	3501 (1.2)	3424 (1.4)	3839 (1.6)	0.01
**Unspecified chronic bronchitis**	3984 (1.4)	3905 (1.6)	4428 (1.8)	0.02
**Nicotine dependence; n (%)**	28443 (9.8)	26802 (11.1)	27042 (11.2)	0.003
**Psychiatric comorbidities; n (%)**				
**Anxiety disorders**	71009 (24.4)	66055 (27.3)	65518 (27.1)	0.005
**Substance misuse**	40768 (14.0)	38056 (15.7)	38315 (15.8)	0.003

**Mood disorders**	63121 (21.7)	57322 (23.7)	56855 (23.5)	0.005
**Psychotic disorders**	7557 (2.6)	6191 (2.6)	6248 (2.6)	0.001
**Heart disease; n (%)**				
**Ischemic heart diseases**	87155 (29.9)	75509 (31.2)	75164 (31.0)	0.003
**Other forms of heart disease**	125123 (43.0)	109846 (45.4)	108748 (44.9)	0.009
**Chronic kidney diseases; n (%)**				
**Chronic kidney disease (CKD)**	62598 (21.5)	53852 (22.2)	53348 (22.0)	0.005
**Hypertensive chronic kidney disease**	33178 (11.4)	27430 (11.3)	27159 (11.2)	0.004
**Chronic liver diseases; n (%)**				
**Alcoholic liver disease**	1781 (0.6)	1586 (0.7)	1610 (0.7)	0.001
**Chronic hepatitis, not elsewhere classified**	641 (0.2)	617 (0.3)	648 (0.3)	0.003
**Chronic passive congestion of liver**	3040 (1.0)	2834 (1.2)	2942 (1.2)	0.004
**Fatty (change of) liver, not elsewhere classified**	13877 (4.8)	12214 (5.0)	12235 (5.1)	0.0004
**Fibrosis and cirrhosis of liver**	6573 (2.3)	5765 (2.4)	5901 (2.4)	0.004
**Hepatic failure, not elsewhere classified**	3458 (1.2)	3032 (1.3)	3173 (1.3)	0.005
**Other specified diseases of liver**	11228 (3.9)	10293 (4.3)	10424 (4.3)	0.003
**Portal hypertension**	2398 (0.8)	2107 (0.9)	2142 (0.9)	0.002
**Cerebral infarction; n (%)**	23700 (8.1)	20233 (8.4)	20359 (8.4)	0.002
**Dementia; n (%)**				
**Alzheimer's disease**	7164 (2.5)	5004 (2.1)	4948 (2.0)	0.002
**Dementia in other diseases classified elsewhere**	8989 (3.1)	6345 (2.6)	6292 (2.6)	0.001
**Dementia with Lewy bodies**	730 (0.3)	548 (0.2)	568 (0.2)	0.002
**Frontotemporal dementia**	389 (0.1)	280 (0.1)	287 (0.1)	0.0008
**Unspecified dementia**	19787 (6.8)	14716 (6.1)	14640 (6.0)	0.001
**Vascular dementia**	4557 (1.6)	3418 (1.4)	3384 (1.4)	0.001
**Neoplasms; n (%)**				
**Malignant neoplasms of lymphoid, hematopoietic and related tissue**	8956 (3.1)	8459 (3.5)	8335 (3.4)	0.003
**Neoplasms (benign or malignant)**	105905 (36.4)	98128 (40.5)	96552 (39.9)	0.01
**Organ transplant; n (%)**				
**Liver Transplantation Procedures**	252 (0.1)	207 (0.1)	202 (0.1)	0.0007
**Renal Transplantation Procedures**	757 (0.3)	521 (0.2)	534 (0.2)	0.001
**Psoriasis; n (%)**	5753 (2.0)	5326 (2.2)	5328 (2.2)	0.00006
**Rheumatoid arthritis; n (%)**				
**Other rheumatoid arthritis**	10662 (3.7)	9768 (4.0)	9902 (4.1)	0.003
**Rheumatoid arthritis with rheumatoid factor**	2885 (1.0)	2686 (1.1)	2777 (1.1)	0.004
**Systemic lupus erythematosus (SLE); n (%)**	1918 (0.7)	1797 (0.7)	1833 (0.8)	0.002
**Disorders involving the immune mechanism; n (%)**	12102 (4.2)	10976 (4.5)	11181 (4.6)	0.004
**MEDICATIONS**				
**COVID-19 vaccine; n (%)**				
**Pfizer**	6819 (2.3)	6489 (2.7)	6652 (2.7)	0.004
**Moderna**	1414 (0.5)	1369 (0.6)	1483 (0.6)	0.006
**Janssen**	170 (0.1)	166 (0.1)	164 (0.1)	0.0003
**Not specified**	10520 (3.6)	10010 (4.1)	10211 (4.2)	0.004

**Antidepressants; n (%)**				
**Any**	88326 (30.3)	80196 (33.1)	79971 (33.0)	0.002
**Fluvoxamine**	415 (0.1)	336 (0.1)	325 (0.1)	0.001
**Lithium; n (%)**	618 (0.2)	525 (0.2)	547 (0.2)	0.002
**Antipsychotics; n (%)**				
**Any**	31625 (10.9)	26822 (11.1)	26520 (11.0)	0.004
**Clozapine**	230 (0.1)	172 (0.1)	187 (0.1)	0.002
**INDEX EVENT; n (%)**				
**Feb-Mar 2020**	62196 (21.4)	36372 (15)	36372 (15)	0
**Apr-May 2020**	761 (0.3)	733 (0.3)	733 (0.3)	0
**Jun-Jul 2020**	15229 (5.2)	14248 (5.9)	14248 (5.9)	0
**Aug-Sep 2020**	14478 (5)	13543 (5.6)	13543 (5.6)	0
**Oct-Nov 2020**	15072 (5.2)	14479 (6)	14479 (6)	0
**Dec-Jan 2021**	27915 (9.6)	25374 (10.5)	25374 (10.5)	0
**Feb-Mar 2021**	46792 (16.1)	39475 (16.3)	39475 (16.3)	0
**Apr-May 2021**	26885 (9.2)	22895 (9.5)	22895 (9.5)	0
**Jun-Jul 2021**	13761 (4.7)	13551 (5.6)	13551 (5.6)	0
**Aug-Sep 2021**	7857 (2.7)	7849 (3.2)	7849 (3.2)	0
**Oct-Nov 2021**	32488 (11.2)	28036 (11.6)	28036 (11.6)	0
**Dec-Jan 2022**	27679 (9.5)	25546 (10.6)	25546 (10.6)	0

Table S5 – Uncorrected p-values and Bonferroni-corrected p-values for the 6-month constant HRs comparing risks after COVID-19 vs. other respiratory infection in the whole cohort.

Outcome	HR	p	p (corrected)
Anxiety disorder	1.13 (1.11-1.15)	< 0.0001	<0.0001
Cognitive deficit	1.36 (1.33-1.39)	< 0.0001	<0.0001
Dementia	1.33 (1.26-1.41)	< 0.0001	<0.0001
Encephalitis	0.96 (0.85-1.08)	0.5	1
Epilepsy or seizures	1.14 (1.09-1.19)	< 0.0001	<0.0001
GBS	1.12 (0.97-1.30)	0.12	1
Insomnia	1.13 (1.10-1.16)	< 0.0001	<0.0001
Intracranial haemorrhage	1.09 (1.01-1.18)	0.02	0.29
Ischaemic stroke	1.11 (1.06-1.17)	< 0.0001	0.00014
Mood disorder	1.08 (1.06-1.11)	< 0.0001	<0.0001
Myoneural junction/muscle disease	1.89 (1.76-2.04)	< 0.0001	<0.0001
Nerve/Nerve root/Plexus disorder	0.89 (0.87-0.91)	< 0.0001	<0.0001
Parkinsonism	1.04 (0.92-1.17)	0.58	1
Psychotic disorder	1.27 (1.18-1.37)	< 0.0001	<0.0001
Any first	1.13 (1.11-1.15)	< 0.0001	<0.0001

Table S6 – Markers of severity of the infection occurring within 14 days of the index infection in the matched cohorts. SMD=Standardised mean difference. Percentages in brackets.

Severity marker	COVID-19	Other respiratory infections	SMD
Hospitalisation	130688 (10.2)	71801 (5.6)	0.17
ICU	23967 (1.9)	14294 (1.1)	0.062
Mechanical Ventilation	25835 (2.0)	13732 (1.1)	0.077

Table S7 – Death rate (in %) within the first 6 months following a diagnosis of COVID-19 or other respiratory infection in matched cohorts for different age groups. The higher death rate in COVID-19 indicates that death is a competing risk which brings higher HRs closer to 1.

Age group	COVID-19	Other respiratory infection
Children/Adolescents	0.15 (0.12-0.18)	0.11 (0.091-0.13)
Adults	1.84 (1.80-1.87)	1.51 (1.48-1.54)
Older adults	10.82 (10.67-10.97)	8.08 (7.95-8.21)
All	3.56 (3.52-3.60)	2.74 (2.70-2.77)

Table S8 – HR (and 95% CI) for the risk of other outcomes not presented in the main manuscript (those including first and recurrent diagnoses (denoted by "(any)") and the composite of individual outcomes or death), within 6 months of a diagnosis of COVID-19 vs. another respiratory infection. GBS=Guillain-Barré Syndrome.

Outcome	All	Children	Adults	Older Adults
Anxiety disorder (any)	1.05 (1.04-1.06)	1.11 (1.07-1.15)	1.05 (1.04-1.06)	1.00 (0.98-1.02)
Cognitive deficit (any)	1.13 (1.11-1.14)	1.22 (1.14-1.31)	1.10 (1.08-1.12)	1.16 (1.14-1.18)
Epilepsy or seizures (any)	1.06 (1.04-1.08)	1.44 (1.35-1.52)	1.01 (0.98-1.04)	1.03 (0.99-1.07)
Insomnia (any)	1.01 (0.99-1.02)	1.23 (1.11-1.37)	1.00 (0.98-1.02)	1.01 (0.98-1.03)
Intracranial haemorrhage (any)	0.92 (0.87-0.96)	1.63 (1.30-2.05)	0.86 (0.80-0.92)	0.94 (0.87-1.00)
Ischaemic stroke (any)	0.94 (0.92-0.97)	1.30 (0.98-1.72)	0.93 (0.89-0.97)	0.95 (0.92-0.99)
Mood disorder (any)	1.03 (1.02-1.04)	1.13 (1.07-1.18)	1.03 (1.02-1.04)	1.02 (1.01-1.04)
Psychotic disorder (any)	1.13 (1.09-1.17)	2.39 (1.73-3.30)	1.10 (1.05-1.14)	1.12 (1.04-1.20)
Any	1.04 (1.03-1.04)	1.18 (1.15-1.21)	1.03 (1.02-1.04)	1.03 (1.02-1.04)
Anxiety disorder or Death	1.23 (1.21-1.24)	1.02 (0.96-1.08)	1.18 (1.16-1.20)	1.33 (1.30-1.36)
Cognitive deficit or Death	1.48 (1.46-1.51)	1.21 (1.10-1.33)	1.45 (1.41-1.48)	1.56 (1.53-1.60)
Dementia or Death	1.40 (1.38-1.43)	1.41 (1.11-1.80)	1.29 (1.25-1.32)	1.49 (1.46-1.52)
Encephalitis or Death	1.37 (1.34-1.39)	1.45 (1.18-1.79)	1.27 (1.23-1.30)	1.44 (1.41-1.47)
Epilepsy or seizures or Death	1.41 (1.38-1.43)	1.44 (1.26-1.63)	1.32 (1.28-1.36)	1.47 (1.44-1.50)
GBS or Death	1.37 (1.35-1.40)	1.45 (1.14-1.84)	1.28 (1.24-1.31)	1.44 (1.41-1.47)
Insomnia or Death	1.30 (1.28-1.32)	1.32 (1.17-1.49)	1.22 (1.19-1.24)	1.38 (1.35-1.41)
Intracranial haemorrhage or Death	1.39 (1.37-1.42)	1.56 (1.26-1.94)	1.29 (1.25-1.33)	1.46 (1.43-1.50)
Ischaemic stroke or Death	1.39 (1.37-1.42)	1.57 (1.25-1.98)	1.31 (1.27-1.35)	1.45 (1.42-1.49)
Mood disorder or Death	1.25 (1.23-1.27)	1.04 (0.96-1.12)	1.16 (1.14-1.18)	1.39 (1.36-1.42)
Myoneural junction/muscle disease or Death	1.41 (1.38-1.43)	1.52 (1.22-1.90)	1.34 (1.30-1.38)	1.46 (1.43-1.49)
Nerve/Nerve root/Plexus disorder or Death	1.18 (1.17-1.20)	1.41 (1.18-1.68)	1.08 (1.05-1.10)	1.29 (1.27-1.32)
Parkinsonism or Death	1.38 (1.35-1.40)	1.42 (1.11-1.81)	1.28 (1.24-1.31)	1.44 (1.41-1.47)
Psychotic disorder or Death	1.38 (1.36-1.40)	1.51 (1.21-1.87)	1.28 (1.24-1.31)	1.45 (1.42-1.48)
Any first or Death	1.23 (1.21-1.24)	1.04 (0.99-1.10)	1.17 (1.15-1.19)	1.39 (1.35-1.42)
Anxiety disorder (any) or Death	1.10 (1.09-1.11)	1.12 (1.08-1.16)	1.08 (1.07-1.09)	1.16 (1.15-1.18)
Cognitive deficit (any) or Death	1.23 (1.22-1.25)	1.23 (1.15-1.32)	1.18 (1.16-1.20)	1.29 (1.27-1.31)
Epilepsy or seizures (any) or Death	1.25 (1.24-1.27)	1.43 (1.35-1.51)	1.14 (1.12-1.16)	1.36 (1.33-1.38)
Insomnia (any) or Death	1.17 (1.15-1.18)	1.24 (1.13-1.37)	1.08 (1.07-1.10)	1.27 (1.25-1.29)
Intracranial haemorrhage (any) or Death	1.33 (1.30-1.35)	1.51 (1.27-1.79)	1.21 (1.18-1.25)	1.40 (1.38-1.43)
Ischaemic stroke (any) or Death	1.24 (1.22-1.26)	1.34 (1.12-1.62)	1.16 (1.13-1.19)	1.30 (1.28-1.33)
Mood disorder (any) or Death	1.10 (1.10-1.11)	1.14 (1.09-1.20)	1.06 (1.05-1.07)	1.19 (1.17-1.21)
Psychotic disorder (any) or Death	1.33 (1.31-1.35)	1.68 (1.38-2.03)	1.22 (1.19-1.25)	1.41 (1.39-1.44)
Any or Death	1.07 (1.06-1.07)	1.18 (1.15-1.22)	1.05 (1.04-1.05)	1.10 (1.09-1.11)

Table S9 – Summary of the risk trajectories in children for the comparison between COVID-19 and other respiratory infections: HRs at 6 months with the corresponding uncorrected and Bonferroni-corrected p-values, risk horizon and time to equal incidence within 2 years of infection.

Outcome	HR (6 months)	p	p (corrected)	Risk horizon [days]	Time to equal incidence [days]
Anxiety disorder	1.00 (0.94-1.06)	0.98	1	-	-
Cognitive deficit	1.20 (1.09-1.33)	0.00028	0.0039	75	491
Dementia	1.41 (0.45-4.45)	0.55	1	-	-
Encephalitis	1.79 (1.21-2.66)	0.0032	0.045	>730	>730
Epilepsy or seizures	1.44 (1.25-1.65)	< 0.0001	<0.0001	>730	>730
GBS	2.20 (0.88-5.51)	0.084	1	-	-
Insomnia	1.29 (1.12-1.48)	0.00033	0.0046	92	405
Intracranial haemorrhage	2.16 (1.46-3.19)	< 0.0001	0.0011	>730	>730
Ischaemic stroke	1.89 (1.15-3.09)	0.0099	0.14	35	>730
Mood disorder	1.02 (0.94-1.10)	0.63	1	-	-
Myoneural junction/muscle disease	1.90 (1.19-3.02)	0.0062	0.086	>730	>730
Nerve/Nerve root/Plexus disorder	1.39 (1.08-1.78)	0.01	0.14	>730	>730
Parkinsonism	0.99 (0.14-7.04)	0.99	1	-	-
Psychotic disorder	2.00 (1.26-3.19)	0.0029	0.04	75	>730
Any first	1.03 (0.98-1.09)	0.22	1	-	-

Table S10 – Summary of the risk trajectories in adults for the comparison between COVID-19 and other respiratory infections: HRs at 6 months with the corresponding uncorrected and Bonferroni-corrected p-values, risk horizon and time to equal incidence within 2 years of infection.

**Outcome**	**HR (6 months)**	**p**	**p (corrected)**	**Risk horizon [days]**	**Time to equal incidence [days]**
Anxiety disorder	1.13 (1.11-1.15)	< 0.0001	<0.0001	65	466
Cognitive deficit	1.35 (1.31-1.40)	< 0.0001	<0.0001	>730	>730
Dementia	1.14 (0.98-1.33)	0.087	1	-	-
Encephalitis	0.87 (0.75-1.01)	0.062	0.86	-	-
Epilepsy or seizures	1.08 (1.02-1.14)	0.013	0.18	50	>730
GBS	1.08 (0.89-1.30)	0.46	1	-	-
Insomnia	1.11 (1.07-1.14)	< 0.0001	<0.0001	84	603
Intracranial haemorrhage	0.98 (0.88-1.10)	0.75	1	-	-
Ischaemic stroke	1.12 (1.05-1.21)	0.0012	0.017	100	588
Mood disorder	1.06 (1.04-1.09)	< 0.0001	<0.0001	44	330
Myoneural junction/muscle disease	1.88 (1.71-2.07)	< 0.0001	<0.0001	>730	>730
Nerve/Nerve root/Plexus disorder	0.91 (0.89-0.94)	< 0.0001	<0.0001	-	-
Parkinsonism	0.85 (0.66-1.11)	0.23	1	-	-
Psychotic disorder	1.18 (1.08-1.29)	0.00029	0.0041	86	657
Any first	1.11 (1.09-1.13)	< 0.0001	<0.0001	48	384

Table S11 - Uncorrected p-values and Bonferroni-corrected p-values for the 6-month constant HRs comparing risks after COVID-19 vs. other respiratory infection in older adults.

Outcome	HR (6 months)	p	p (corrected)	Risk horizon [days]	Time to equal incidence [days]
Anxiety disorder	1.16 (1.11-1.20)	< 0.0001	<0.0001	44	463
Cognitive deficit	1.41 (1.36-1.46)	< 0.0001	<0.0001	>730	>730
Dementia	1.41 (1.33-1.50)	< 0.0001	<0.0001	>730	>730
Encephalitis	0.97 (0.78-1.22)	0.82	1	-	-
Epilepsy or seizures	1.17 (1.08-1.26)	0.00011	0.0016	91	>730
GBS	1.10 (0.87-1.39)	0.41	1	-	-
Insomnia	1.16 (1.10-1.22)	< 0.0001	<0.0001	116	>730
Intracranial haemorrhage	1.15 (1.03-1.28)	0.01	0.14	0	23
Ischaemic stroke	1.11 (1.04-1.18)	0.0015	0.022	>730	>730
Mood disorder	1.17 (1.11-1.22)	< 0.0001	<0.0001	36	>730
Myoneural junction/muscle disease	1.82 (1.61-2.05)	< 0.0001	<0.0001	247	723
Nerve/Nerve root/Plexus disorder	0.85 (0.81-0.89)	< 0.0001	<0.0001	-	-
Parkinsonism	1.16 (1.01-1.33)	0.041	0.58	>730	692
Psychotic disorder	1.39 (1.21-1.59)	< 0.0001	<0.0001	>730	>730
Any first	1.22 (1.19-1.26)	< 0.0001	<0.0001	50	>730

Table S12 – 2-year cumulative incidence (with 95% CI) in the matched COVID-19 and other respiratory infection cohorts and the p-values (derived from bootstrap with 1000 repetitions) for the test of the null hypothesis that no difference in incidence exists between cohorts. This corresponds to the number presented in Fig. 3 of the main manuscript.

	Children			Adults			Older adults		
	COVID-19 incidence [%]	Other resp. inf. incidence [%]	p-value	COVID-19 incidence [%]	Other resp. inf. incidence [%]	p-value	COVID-19 incidence [%]	Other resp. inf. incidence [%]	p-value
**Anxiety disorder**	10.82 (9.47-12.16)	13.67 (11.44-15.84)	0.0060	18.23 (16.58-19.85)	18.77 (17.85-19.68)	0.29	11.74 (10.97-12.50)	11.58 (10.94-12.22)	0.40
**Cognitive deficit**	3.92 (3.18-4.66)	5.04 (3.07-6.97)	0.14	6.39 (5.88-6.89)	5.50 (5.12-5.88)	0.0040	15.44 (14.06-16.81)	12.31 (11.61-13.01)	0.0010
**Dementia**	0.013 (0.0044-0.021)	0.0028 (0.00035-0.0053)	0.010	0.20 (0.17-0.23)	0.22 (0.18-0.26)	0.23	4.46 (4.19-4.73)	3.34 (3.08-3.61)	0.0010
**Encephalitis**	0.076 (0.056-0.096)	0.051 (0.035-0.067)	0.022	0.11 (0.096-0.13)	0.12 (0.10-0.14)	0.27	0.14 (0.11-0.16)	0.14 (0.12-0.17)	0.34
**Epilepsy or seizures**	2.63 (1.35-3.89)	1.26 (1.04-1.47)	0.0010	1.77 (1.15-2.38)	1.36 (1.19-1.54)	0.061	1.98 (1.83-2.13)	1.79 (1.62-1.97)	0.065
**GBS**	0.0099 (0.0046-0.015)	0.015 (0.0044-0.025)	0.19	0.061 (0.052-0.071)	0.052 (0.044-0.061)	0.092	0.23 (0.022-0.43)	0.099 (0.082-0.12)	0.022
**Insomnia**	1.58 (1.33-1.83)	1.54 (1.20-1.87)	0.43	6.09 (5.65-6.52)	5.91 (5.60-6.23)	0.26	7.10 (6.62-7.58)	6.81 (6.31-7.30)	0.25
**Intracranial haemorrhage**	0.12 (0.085-0.16)	0.091 (0.049-0.13)	0.16	0.39 (0.30-0.49)	0.35 (0.30-0.39)	0.23	1.08 (0.97-1.20)	1.15 (0.87-1.44)	0.42
**Ischaemic stroke**	0.11 (0.057-0.16)	0.10 (0.051-0.15)	0.40	1.08 (0.95-1.21)	1.20 (0.99-1.41)	0.20	4.11 (3.78-4.44)	3.83 (3.40-4.26)	0.20
**Mood disorder**	6.39 (5.06-7.69)	6.78 (5.85-7.71)	0.34	11.56 (10.17-12.93)	11.48 (10.87-12.09)	0.50	9.51 (8.17-10.84)	8.93 (8.04-9.81)	0.15
**Myoneural junction/muscle disease**	0.11 (0.074-0.14)	0.057 (0.038-0.075)	0.0040	0.44 (0.40-0.48)	0.32 (0.28-0.36)	0.001	0.79 (0.70-0.89)	0.84 (0.47-1.20)	0.44
**Nerve/Nerve root/Plexus disorder**	0.41 (0.28-0.55)	0.27 (0.19-0.34)	0.023	6.84 (5.92-7.76)	6.87 (6.50-7.24)	0.48	7.96 (7.24-8.67)	7.89 (7.43-8.35)	0.48
**Parkinsonism**	0.0038 (0.00-0.0079)	0.0011 (0.00-0.0027)	0.11	0.059 (0.048-0.07)	0.19 (0.00-0.41)	0.018	0.71 (0.62-0.80)	0.80 (0.60-1.00)	0.20
**Psychotic disorder**	0.18 (0.096-0.27)	0.063 (0.037-0.09)	0.0020	0.51 (0.46-0.56)	0.57 (0.49-0.65)	0.12	0.85 (0.71-0.98)	0.60 (0.51-0.68)	0.001
**Any first**	19.67 (16.49-22.74)	21.11 (17.62-24.45)	0.29	29.17 (26.62-31.63)	29.11 (27.97-30.23)	0.47	31.18 (29.30-33.02)	30.07 (28.07-32.01)	0.24

Table S13 – Incidence of subcategories of diagnoses within 2 years of a diagnosis of COVID-19 (within the unmatched COVID-19 cohort)

Outcome	Incidence [%]
**Cognitive deficit**	
MCI (G51.84)	0.7
Somnolence/Stupor/Coma (R40)	4.59
Other cognitive symptoms (R41)	11.82
Symbolic dysfunction (R48)	0.53
Delirium (F05)	0.83
Encephalopathy (G93.40)	2.32
**Nerve/Nerve root/Plexus disorder**	
Cranial nerve disorder (G50-G53)	1.1
Nerve root and plexus disorder (G54-G55)	0.61
Mononeuropathies (G56-G59)	6.4
**Myoneural junction/muscle disease**	
Myasthenia gravis (G70.0)	0.063
Myoneural disorder; unspecified (G70.9)	0.18
Primary disorders of muscles (G71)	0.08
Specified myopathies (G72)	0.34
Critical illness myopathy and other specified myopathies (G72.8)	0.2
Myopathy; unspecified (G72.9)	0.084
Myoneural junction/muscle disorder in diseases classified elsewhere (G73)	0.0077
**Epilepsy or Seizures**	
Epilepsy (G40)	1.2
Seizures (R56)	5.75

Table S14 - All baseline characteristics for the cohorts diagnosed with COVID-19 after and before the emergence of the alpha variant. A baseline characteristic with a standardized mean difference (SMD) less than 0.1 is considered well matched.

	COVID (alpha)	COVID (pre-alpha)	SMD
**Number**	47675	47675	
**DEMOGRAPHICS**			
**Age; mean (SD)**	44.4 (20.7)	44.5 (21.3)	0.005
**Sex; n (%)**			
**Female**	26169 (54.9)	26117 (54.8)	0.002
**Male**	21491 (45.1)	21544 (45.2)	0.002
**Other**	15 (0.0)	14 (0.0)	0.001
**Race; n (%)**			
**White**	26802 (56.2)	26825 (56.3)	0.001
**Black or African American**	8781 (18.4)	8864 (18.6)	0.004
**Asian**	1050 (2.2)	1045 (2.2)	0.0007
**American Indian or Alaska Native**	164 (0.3)	165 (0.3)	0.0004
**Native Hawaiian or Other Pacific Islander**	62 (0.1)	67 (0.1)	0.003
**Unknown**	10816 (22.7)	10709 (22.5)	0.005
**Ethnicity; n (%)**			
**Hispanic or Latino**	4725 (9.9)	4684 (9.8)	0.003
**Not Hispanic or Latino**	30123 (63.2)	30162 (63.3)	0.002
**Unknown**	12827 (26.9)	12829 (26.9)	0.00009
**Problems related to housing and economic circumstances; n (%)**	1105 (2.3)	1073 (2.3)	0.004
**COMORBIDITIES**			
**Overweight and obesity; n (%)**	11394 (23.9)	11251 (23.6)	0.007
**Hypertensive disease; n (%)**			
**Hypertensive diseases**	15731 (33.0)	15585 (32.7)	0.007
**Hypertensive diseases (deprecated 2018)**	15717 (33.0)	15572 (32.7)	0.006
**Diabetes mellitus; n (%)**			
**Type 1 diabetes mellitus**	1026 (2.2)	1023 (2.1)	0.0004
**Type 2 diabetes mellitus**	7859 (16.5)	7824 (16.4)	0.002
**Chronic lower respiratory diseases; n (%)**			
**Asthma**	6574 (13.8)	6534 (13.7)	0.002
**Bronchiectasis**	228 (0.5)	240 (0.5)	0.004
**Bronchitis, not specified as acute or chronic**	2834 (5.9)	2832 (5.9)	0.0002
**Emphysema**	925 (1.9)	900 (1.9)	0.004
**Other chronic obstructive pulmonary disease**	2657 (5.6)	2593 (5.4)	0.006
**Simple and mucopurulent chronic bronchitis**	207 (0.4)	199 (0.4)	0.003
**Unspecified chronic bronchitis**	272 (0.6)	273 (0.6)	0.0003
**Nicotine dependence; n (%)**	5379 (11.3)	5283 (11.1)	0.006
**Psychiatric comorbidities; n (%)**			
**Anxiety disorders**	11265 (23.6)	11131 (23.3)	0.007
**Substance misuse**	7255 (15.2)	7116 (14.9)	0.008

**Mood disorders**	8877 (18.6)	8704 (18.3)	0.009
**Psychotic disorders**	748 (1.6)	763 (1.6)	0.003
**Heart disease; n (%)**			
**Ischemic heart diseases**	4930 (10.3)	4879 (10.2)	0.004
**Other forms of heart disease**	9262 (19.4)	9115 (19.1)	0.008
**Chronic kidney diseases; n (%)**			
**Chronic kidney disease (CKD)**	3695 (7.8)	3672 (7.7)	0.002
**Hypertensive chronic kidney disease**	1824 (3.8)	1815 (3.8)	0.001
**Chronic liver diseases; n (%)**			
**Alcoholic liver disease**	225 (0.5)	235 (0.5)	0.003
**Chronic hepatitis, not elsewhere classified**	60 (0.1)	59 (0.1)	0.0006
**Chronic passive congestion of liver**	275 (0.6)	268 (0.6)	0.002
**Fatty (change of) liver, not elsewhere classified**	2010 (4.2)	2002 (4.2)	0.0008
**Fibrosis and cirrhosis of liver**	583 (1.2)	598 (1.3)	0.003
**Hepatic failure, not elsewhere classified**	306 (0.6)	322 (0.7)	0.004
**Other specified diseases of liver**	1080 (2.3)	1053 (2.2)	0.004
**Portal hypertension**	222 (0.5)	233 (0.5)	0.003
**Cerebral infarction; n (%)**	1562 (3.3)	1538 (3.2)	0.003
**Dementia; n (%)**			
**Alzheimer's disease**	183 (0.4)	199 (0.4)	0.005
**Dementia in other diseases classified elsewhere**	222 (0.5)	230 (0.5)	0.002
**Dementia with Lewy bodies**	12 (0.0)	10 (0.0)	0.003
**Frontotemporal dementia**	10 (0.0)	10 (0.0)	0
**Unspecified dementia**	536 (1.1)	528 (1.1)	0.002
**Vascular dementia**	121 (0.3)	124 (0.3)	0.001
**Neoplasms; n (%)**			
**Malignant neoplasms of lymphoid, hematopoietic and related tissue**	661 (1.4)	648 (1.4)	0.002
**Neoplasms (benign or malignant)**	9868 (20.7)	9794 (20.5)	0.004
**Organ transplant; n (%)**			
**Liver Transplantation Procedures**	13 (0.0)	12 (0.0)	0.001
**Renal Transplantation Procedures**	88 (0.2)	95 (0.2)	0.003
**Psoriasis; n (%)**	632 (1.3)	609 (1.3)	0.004
**Rheumatoid arthritis; n (%)**			
**Other rheumatoid arthritis**	862 (1.8)	871 (1.8)	0.001
**Rheumatoid arthritis with rheumatoid factor**	225 (0.5)	227 (0.5)	0.0006
**Systemic lupus erythematosus (SLE); n (%)**	310 (0.7)	312 (0.7)	0.0005
**Disorders involving the immune mechanism; n (%)**	1405 (2.9)	1395 (2.9)	0.001
**MEDICATIONS**			
**COVID-19 vaccine; n (%)**			
**Pfizer**	345 (0.7)	332 (0.7)	0.003
**Moderna**	56 (0.1)	58 (0.1)	0.001
**Janssen**	10 (0.0)	10 (0.0)	0
**Not specified**	366 (0.8)	396 (0.8)	0.007

**Antidepressants; n (%)**			
**Any**	10967 (23.0)	10880 (22.8)	0.004
**Fluvoxamine**	64 (0.1)	64 (0.1)	0
**Lithium; n (%)**	139 (0.3)	141 (0.3)	0.0008
**Antipsychotics; n (%)**			
**Any**	3287 (6.9)	3260 (6.8)	0.002
**Clozapine**	18 (0.0)	21 (0.0)	0.003

Table S15 - All baseline characteristics for the cohorts diagnosed with COVID-19 after and before the emergence of the delta variant. A baseline characteristic with a standardized mean difference (SMD) less than 0.1 is considered well matched.

	COVID (delta)	COVID (pre-delta)	SMD
**Number**	44835	44835	
**DEMOGRAPHICS**			
**Age; mean (SD)**	43.3 (21.2)	43.1 (21.0)	0.008
**Sex; n (%)**			
**Female**	24899 (55.5)	25003 (55.8)	0.005
**Male**	19922 (44.4)	19818 (44.2)	0.005
**Other**	14 (0.0)	14 (0.0)	0
**Race; n (%)**			
**White**	23052 (51.4)	23061 (51.4)	0.0004
**Black or African American**	9091 (20.3)	9202 (20.5)	0.006
**Asian**	868 (1.9)	832 (1.9)	0.006
**American Indian or Alaska Native**	178 (0.4)	174 (0.4)	0.001
**Native Hawaiian or Other Pacific Islander**	61 (0.1)	64 (0.1)	0.002
**Unknown**	11585 (25.8)	11502 (25.7)	0.004
**Ethnicity; n (%)**			
**Hispanic or Latino**	4674 (10.4)	4790 (10.7)	0.008
**Not Hispanic or Latino**	29000 (64.7)	28365 (63.3)	0.03
**Unknown**	11161 (24.9)	11680 (26.1)	0.03
**Problems related to housing and economic circumstances; n (%)**	867 (1.9)	939 (2.1)	0.01
**COMORBIDITIES**			
**Overweight and obesity; n (%)**	10777 (24.0)	10594 (23.6)	0.01
**Hypertensive disease; n (%)**			
**Hypertensive diseases**	14165 (31.6)	14041 (31.3)	0.006
**Hypertensive diseases (deprecated 2018)**	14150 (31.6)	14029 (31.3)	0.006
**Diabetes mellitus; n (%)**			
**Type 1 diabetes mellitus**	917 (2.0)	933 (2.1)	0.003
**Type 2 diabetes mellitus**	7003 (15.6)	6995 (15.6)	0.0005
**Chronic lower respiratory diseases; n (%)**			
**Asthma**	6138 (13.7)	6107 (13.6)	0.002
**Bronchiectasis**	258 (0.6)	259 (0.6)	0.0003
**Bronchitis, not specified as acute or chronic**	2665 (5.9)	2652 (5.9)	0.001
**Emphysema**	751 (1.7)	813 (1.8)	0.01
**Other chronic obstructive pulmonary disease**	2252 (5.0)	2308 (5.1)	0.006
**Simple and mucopurulent chronic bronchitis**	186 (0.4)	184 (0.4)	0.0007
**Unspecified chronic bronchitis**	259 (0.6)	259 (0.6)	0
**Nicotine dependence; n (%)**	5287 (11.8)	5299 (11.8)	0.0008
**Psychiatric comorbidities; n (%)**			
**Anxiety disorders**	10642 (23.7)	10670 (23.8)	0.001
**Substance misuse**	7044 (15.7)	7128 (15.9)	0.005

**Mood disorders**	8245 (18.4)	8336 (18.6)	0.005
**Psychotic disorders**	722 (1.6)	753 (1.7)	0.005
**Heart disease; n (%)**			
**Ischemic heart diseases**	4440 (9.9)	4429 (9.9)	0.0008
**Other forms of heart disease**	8923 (19.9)	8854 (19.7)	0.004
**Chronic kidney diseases; n (%)**			
**Chronic kidney disease (CKD)**	3295 (7.3)	3348 (7.5)	0.005
**Hypertensive chronic kidney disease**	1714 (3.8)	1708 (3.8)	0.0007
**Chronic liver diseases; n (%)**			
**Alcoholic liver disease**	208 (0.5)	216 (0.5)	0.003
**Chronic hepatitis, not elsewhere classified**	61 (0.1)	54 (0.1)	0.004
**Chronic passive congestion of liver**	225 (0.5)	225 (0.5)	0
**Fatty (change of) liver, not elsewhere classified**	1877 (4.2)	1906 (4.3)	0.003
**Fibrosis and cirrhosis of liver**	528 (1.2)	543 (1.2)	0.003
**Hepatic failure, not elsewhere classified**	337 (0.8)	336 (0.7)	0.0003
**Other specified diseases of liver**	1036 (2.3)	1047 (2.3)	0.002
**Portal hypertension**	239 (0.5)	242 (0.5)	0.0009
**Cerebral infarction; n (%)**	1379 (3.1)	1392 (3.1)	0.002
**Dementia; n (%)**			
**Alzheimer's disease**	142 (0.3)	152 (0.3)	0.004
**Dementia in other diseases classified elsewhere**	181 (0.4)	197 (0.4)	0.006
**Dementia with Lewy bodies**	15 (0.0)	18 (0.0)	0.003
**Frontotemporal dementia**	10 (0.0)	10 (0.0)	0
**Unspecified dementia**	471 (1.1)	500 (1.1)	0.006
**Vascular dementia**	109 (0.2)	109 (0.2)	0
**Neoplasms; n (%)**			
**Malignant neoplasms of lymphoid, hematopoietic and related tissue**	624 (1.4)	627 (1.4)	0.0006
**Neoplasms (benign or malignant)**	9311 (20.8)	9076 (20.2)	0.01
**Organ transplant; n (%)**			
**Liver Transplantation Procedures**	20 (0.0)	23 (0.1)	0.003
**Renal Transplantation Procedures**	91 (0.2)	85 (0.2)	0.003
**Psoriasis; n (%)**	558 (1.2)	558 (1.2)	0
**Rheumatoid arthritis; n (%)**			
**Other rheumatoid arthritis**	763 (1.7)	767 (1.7)	0.0007
**Rheumatoid arthritis with rheumatoid factor**	226 (0.5)	222 (0.5)	0.001
**Systemic lupus erythematosus (SLE); n (%)**	314 (0.7)	317 (0.7)	0.0008
**Disorders involving the immune mechanism; n (%)**	1421 (3.2)	1406 (3.1)	0.002
**MEDICATIONS**			
**COVID-19 vaccine; n (%)**			
**Pfizer**	636 (1.4)	614 (1.4)	0.004
**Moderna**	79 (0.2)	75 (0.2)	0.002
**Janssen**	25 (0.1)	25 (0.1)	0
**Not specified**	1175 (2.6)	1110 (2.5)	0.009

**Antidepressants; n (%)**			
**Any**	10353 (23.1)	10400 (23.2)	0.002
**Fluvoxamine**	64 (0.1)	59 (0.1)	0.003
**Lithium; n (%)**	122 (0.3)	123 (0.3)	0.0004
**Antipsychotics; n (%)**			
**Any**	3352 (7.5)	3373 (7.5)	0.002
**Clozapine**	14 (0.0)	14 (0.0)	0

Table S16 - All baseline characteristics for the cohorts diagnosed with COVID-19 after and before the emergence of the omicron variant. A baseline characteristic with a standardized mean difference (SMD) less than 0.1 is considered well matched.

	COVID (omicron)	COVID (pre-omicron)	SMD
**Number**	39845	39845	
**DEMOGRAPHICS**			
**Age; mean (SD)**	41.8 (22.3)	41.9 (22.4)	0.007
**Sex; n (%)**			
**Female**	22332 (56.0)	22387 (56.2)	0.003
**Male**	17505 (43.9)	17450 (43.8)	0.003
**Other**	10 (0.0)	10 (0.0)	0
**Race; n (%)**			
**White**	23094 (58.0)	22835 (57.3)	0.01
**Black or African American**	5689 (14.3)	5662 (14.2)	0.002
**Asian**	605 (1.5)	637 (1.6)	0.006
**American Indian or Alaska Native**	107 (0.3)	110 (0.3)	0.001
**Native Hawaiian or Other Pacific Islander**	42 (0.1)	40 (0.1)	0.002
**Unknown**	10308 (25.9)	10561 (26.5)	0.01
**Ethnicity; n (%)**			
**Hispanic or Latino**	3563 (8.9)	3753 (9.4)	0.02
**Not Hispanic or Latino**	27623 (69.3)	27536 (69.1)	0.005
**Unknown**	8659 (21.7)	8556 (21.5)	0.006
**Problems related to housing and economic circumstances; n (%)**	991 (2.5)	960 (2.4)	0.005
**COMORBIDITIES**			
**Overweight and obesity; n (%)**	8289 (20.8)	8334 (20.9)	0.003
**Hypertensive disease; n (%)**			
**Hypertensive diseases**	11409 (28.6)	11593 (29.1)	0.01
**Hypertensive diseases (deprecated 2018)**	11392 (28.6)	11579 (29.1)	0.01
**Diabetes mellitus; n (%)**			
**Type 1 diabetes mellitus**	806 (2.0)	824 (2.1)	0.003
**Type 2 diabetes mellitus**	5422 (13.6)	5517 (13.8)	0.007
**Chronic lower respiratory diseases; n (%)**			
**Asthma**	5910 (14.8)	5821 (14.6)	0.006
**Bronchiectasis**	246 (0.6)	243 (0.6)	0.001
**Bronchitis, not specified as acute or chronic**	2554 (6.4)	2601 (6.5)	0.005
**Emphysema**	759 (1.9)	777 (2.0)	0.003
**Other chronic obstructive pulmonary disease**	2061 (5.2)	2088 (5.2)	0.003
**Simple and mucopurulent chronic bronchitis**	173 (0.4)	177 (0.4)	0.002
**Unspecified chronic bronchitis**	212 (0.5)	209 (0.5)	0.001
**Nicotine dependence; n (%)**	4966 (12.5)	5005 (12.6)	0.003
**Psychiatric comorbidities; n (%)**			
**Anxiety disorders**	10244 (25.7)	10211 (25.6)	0.002
**Substance misuse**	6464 (16.2)	6544 (16.4)	0.005

**Mood disorders**	7555 (19.0)	7597 (19.1)	0.003
**Psychotic disorders**	621 (1.6)	634 (1.6)	0.003
**Heart disease; n (%)**			
**Ischemic heart diseases**	3815 (9.6)	3881 (9.7)	0.006
**Other forms of heart disease**	7811 (19.6)	7910 (19.9)	0.006
**Chronic kidney diseases; n (%)**			
**Chronic kidney disease (CKD)**	2773 (7.0)	2826 (7.1)	0.005
**Hypertensive chronic kidney disease**	1241 (3.1)	1255 (3.1)	0.002
**Chronic liver diseases; n (%)**			
**Alcoholic liver disease**	169 (0.4)	180 (0.5)	0.004
**Chronic hepatitis, not elsewhere classified**	41 (0.1)	42 (0.1)	0.0008
**Chronic passive congestion of liver**	204 (0.5)	215 (0.5)	0.004
**Fatty (change of) liver, not elsewhere classified**	1657 (4.2)	1711 (4.3)	0.007
**Fibrosis and cirrhosis of liver**	440 (1.1)	455 (1.1)	0.004
**Hepatic failure, not elsewhere classified**	222 (0.6)	230 (0.6)	0.003
**Other specified diseases of liver**	935 (2.3)	951 (2.4)	0.003
**Portal hypertension**	156 (0.4)	166 (0.4)	0.004
**Cerebral infarction; n (%)**	1329 (3.3)	1348 (3.4)	0.003
**Dementia; n (%)**			
**Alzheimer's disease**	150 (0.4)	152 (0.4)	0.0008
**Dementia in other diseases classified elsewhere**	185 (0.5)	186 (0.5)	0.0004
**Dementia with Lewy bodies**	17 (0.0)	13 (0.0)	0.005
**Frontotemporal dementia**	10 (0.0)	10 (0.0)	0
**Unspecified dementia**	424 (1.1)	417 (1.0)	0.002
**Vascular dementia**	104 (0.3)	105 (0.3)	0.0005
**Neoplasms; n (%)**			
**Malignant neoplasms of lymphoid, hematopoietic and related tissue**	542 (1.4)	571 (1.4)	0.006
**Neoplasms (benign or malignant)**	8725 (21.9)	8766 (22.0)	0.002
**Organ transplant; n (%)**			
**Liver Transplantation Procedures**	17 (0.0)	16 (0.0)	0.001
**Renal Transplantation Procedures**	62 (0.2)	63 (0.2)	0.0006
**Psoriasis; n (%)**	609 (1.5)	598 (1.5)	0.002
**Rheumatoid arthritis; n (%)**			
**Other rheumatoid arthritis**	776 (1.9)	768 (1.9)	0.001
**Rheumatoid arthritis with rheumatoid factor**	242 (0.6)	243 (0.6)	0.0003
**Systemic lupus erythematosus (SLE); n (%)**	268 (0.7)	270 (0.7)	0.0006
**Disorders involving the immune mechanism; n (%)**	1146 (2.9)	1137 (2.9)	0.001
**MEDICATIONS**			
**COVID-19 vaccine; n (%)**			
**Pfizer**	1087 (2.7)	1032 (2.6)	0.009
**Moderna**	168 (0.4)	177 (0.4)	0.003
**Janssen**	38 (0.1)	40 (0.1)	0.002
**Not specified**	2229 (5.6)	2240 (5.6)	0.001

**Antidepressants; n (%)**			
**Any**	10183 (25.6)	10207 (25.6)	0.001
**Fluvoxamine**	54 (0.1)	53 (0.1)	0.0007
**Lithium; n (%)**	120 (0.3)	123 (0.3)	0.001
**Antipsychotics; n (%)**			
**Any**	3241 (8.1)	3245 (8.1)	0.0004
**Clozapine**	11 (0.0)	14 (0.0)	0.004

Table S17 - Uncorrected p-values and Bonferroni-corrected p-values for the 6-month constant HRs comparing risks in patients diagnosed with COVID-19 after vs. just before the emergence of the alpha variant.

**Outcome**	**HR**	**p**	**p (corrected)**
Anxiety disorder	0.99 (0.91-1.07)	0.77	1
Cognitive deficit	0.93 (0.84-1.03)	0.15	1
Dementia	0.73 (0.57-0.94)	0.014	0.2
Encephalitis	0.81 (0.36-1.85)	0.62	1
Epilepsy or seizures	0.96 (0.79-1.18)	0.72	1
GBS	1.08 (0.57-2.06)	0.81	1
Insomnia	0.94 (0.83-1.07)	0.35	1
Intracranial haemorrhage	0.99 (0.70-1.42)	0.98	1
Ischaemic stroke	0.95 (0.77-1.17)	0.64	1
Mood disorder	1.04 (0.94-1.16)	0.42	1
Myoneural junction/muscle disease	1.01 (0.76-1.35)	0.92	1
Nerve/Nerve root/Plexus disorder	1.17 (1.04-1.31)	0.0085	0.12
Parkinsonism	0.56 (0.27-1.18)	0.12	1
Psychotic disorder	0.94 (0.66-1.34)	0.75	1
Any first	0.98 (0.91-1.05)	0.53	1
Anxiety disorder or Death	0.96 (0.90-1.02)	0.2	1
Cognitive deficit or Death	0.96 (0.89-1.03)	0.23	1
Dementia or Death	0.95 (0.88-1.03)	0.23	1
Epilepsy or seizures or Death	0.98 (0.90-1.06)	0.58	1
Insomnia or Death	0.95 (0.89-1.03)	0.21	1
Intracranial haemorrhage or Death	0.98 (0.91-1.07)	0.72	1
Ischaemic stroke or Death	0.97 (0.90-1.06)	0.54	1
Mood disorder or Death	0.99 (0.92-1.06)	0.82	1
Myoneural junction/muscle disease or Death	1.00 (0.92-1.08)	0.91	1
Nerve/Nerve root/Plexus disorder or Death	1.05 (0.98-1.12)	0.16	1
Psychotic disorder or Death	0.97 (0.90-1.06)	0.54	1
Any first or Death	0.98 (0.92-1.05)	0.58	1

Table S18 - Uncorrected p-values and Bonferroni-corrected p-values for the 6-month constant HRs comparing risks in patients diagnosed with COVID-19 after vs. just before the emergence of the delta variant.

Outcome	HR	p	p (corrected)
Anxiety disorder	1.10 (1.00-1.20)	0.043	0.6
Cognitive deficit	1.13 (1.02-1.26)	0.023	0.32
Dementia	0.60 (0.43-0.84)	0.0023	0.032
Encephalitis	1.22 (0.66-2.27)	0.53	1
Epilepsy or seizures	1.26 (1.00-1.58)	0.048	0.68
GBS	0.68 (0.33-1.39)	0.28	1
Insomnia	1.19 (1.03-1.37)	0.015	0.21
Intracranial haemorrhage	1.11 (0.75-1.65)	0.59	1
Ischaemic stroke	1.27 (1.01-1.60)	0.043	0.6
Mood disorder	0.99 (0.89-1.11)	0.88	1
Myoneural junction/muscle disease	1.19 (0.91-1.56)	0.19	1
Nerve/Nerve root/Plexus disorder	1.04 (0.91-1.18)	0.58	1
Parkinsonism	0.86 (0.41-1.81)	0.69	1
Psychotic disorder	1.15 (0.80-1.64)	0.46	1
Any first	1.08 (1.00-1.17)	0.043	0.61
Anxiety disorder or Death	1.26 (1.18-1.34)	< 0.0001	<0.0001
Cognitive deficit or Death	1.38 (1.27-1.48)	< 0.0001	<0.0001
Dementia or Death	1.49 (1.37-1.62)	< 0.0001	<0.0001
Epilepsy or seizures or Death	1.51 (1.39-1.63)	< 0.0001	<0.0001
Insomnia or Death	1.45 (1.35-1.57)	< 0.0001	<0.0001
Intracranial haemorrhage or Death	1.51 (1.39-1.64)	< 0.0001	<0.0001
Ischaemic stroke or Death	1.51 (1.39-1.64)	< 0.0001	<0.0001
Mood disorder or Death	1.32 (1.23-1.42)	< 0.0001	<0.0001
Myoneural junction/muscle disease or Death	1.52 (1.40-1.64)	< 0.0001	<0.0001
Nerve/Nerve root/Plexus disorder or Death	1.37 (1.28-1.47)	< 0.0001	<0.0001
Psychotic disorder or Death	1.53 (1.41-1.66)	< 0.0001	<0.0001
Any first or Death	1.18 (1.10-1.26)	< 0.0001	<0.0001

Table S19 - Uncorrected p-values and Bonferroni-corrected p-values for the 6-month constant HRs comparing risks in patients diagnosed with COVID-19 after vs. just before the emergence of the omicron variant.

Outcome	HR	p	p (corrected)
Anxiety disorder	1.04 (0.91-1.18)	0.59	1
Cognitive deficit	0.94 (0.81-1.08)	0.39	1
Dementia	1.48 (1.01-2.16)	0.043	0.6
Encephalitis	0.60 (0.25-1.44)	0.24	1
Epilepsy or seizures	0.97 (0.71-1.33)	0.84	1
GBS	0.19 (0.06-0.59)	0.002	0.028
Insomnia	0.95 (0.77-1.16)	0.61	1
Intracranial haemorrhage	1.25 (0.74-2.11)	0.4	1
Ischaemic stroke	0.94 (0.67-1.32)	0.74	1
Mood disorder	1.20 (1.02-1.42)	0.033	0.46
Myoneural junction/muscle disease	0.77 (0.51-1.17)	0.22	1
Nerve/Nerve root/Plexus disorder	1.38 (1.15-1.64)	0.00036	0.0051
Parkinsonism	0.55 (0.24-1.28)	0.15	1
Psychotic disorder	0.96 (0.59-1.58)	0.89	1
Any first	1.03 (0.92-1.15)	0.65	1
Anxiety disorder or Death	0.84 (0.76-0.92)	0.00014	0.002
Cognitive deficit or Death	0.73 (0.66-0.81)	< 0.0001	<0.0001
Dementia or Death	0.69 (0.62-0.78)	< 0.0001	<0.0001
Epilepsy or seizures or Death	0.71 (0.63-0.78)	< 0.0001	<0.0001
Insomnia or Death	0.73 (0.66-0.81)	< 0.0001	<0.0001
Intracranial haemorrhage or Death	0.70 (0.63-0.78)	< 0.0001	<0.0001
Ischaemic stroke or Death	0.71 (0.64-0.79)	< 0.0001	<0.0001
Mood disorder or Death	0.82 (0.74-0.91)	< 0.0001	0.0013
Myoneural junction/muscle disease or Death	0.68 (0.62-0.76)	< 0.0001	<0.0001
Nerve/Nerve root/Plexus disorder or Death	0.82 (0.75-0.90)	< 0.0001	0.00043
Psychotic disorder or Death	0.69 (0.62-0.77)	< 0.0001	<0.0001
Any first or Death	0.89 (0.80-0.98)	0.014	0.19

Table S20 – HR (and 95% CI) for the risk of other outcomes not presented in the main manuscript (those including first and recurrent diagnoses (denoted by "(any)") and the composite of individual outcomes or death), within 6 months of a diagnosis of COVID-19 after vs. before the emergence of specific SARS-CoV-2 variants. GBS=Guillain-Barré Syndrome.

Outcome	Alpha	Delta	Omicron
Anxiety disorder (any)	1.01 (0.97-1.06)	1.05 (1.00-1.10)	1.08 (1.02-1.15)
Cognitive deficit (any)	0.97 (0.91-1.04)	1.04 (0.97-1.12)	1.03 (0.94-1.12)
Epilepsy or seizures (any)	0.94 (0.84-1.04)	1.07 (0.96-1.20)	1.12 (0.98-1.28)
Insomnia (any)	0.96 (0.88-1.03)	1.11 (1.02-1.22)	1.08 (0.96-1.21)
Intracranial haemorrhage (any)	0.84 (0.66-1.06)	1.00 (0.77-1.31)	1.23 (0.88-1.74)
Ischaemic stroke (any)	0.92 (0.81-1.04)	1.06 (0.92-1.22)	1.06 (0.89-1.27)
Mood disorder (any)	1.01 (0.96-1.06)	1.02 (0.97-1.08)	1.19 (1.12-1.28)
Psychotic disorder (any)	0.92 (0.77-1.11)	1.00 (0.83-1.21)	1.20 (0.94-1.53)
Any	0.99 (0.97-1.02)	1.04 (1.01-1.07)	1.08 (1.04-1.13)
Anxiety disorder or Death	0.96 (0.90-1.02)	1.26 (1.18-1.34)	0.84 (0.76-0.92)
Cognitive deficit or Death	0.96 (0.89-1.03)	1.38 (1.27-1.48)	0.73 (0.66-0.81)
Dementia or Death	0.95 (0.88-1.03)	1.49 (1.37-1.62)	0.69 (0.62-0.78)
Encephalitis or Death	0.98 (0.91-1.07)	1.53 (1.41-1.66)	0.68 (0.61-0.76)
Epilepsy or seizures or Death	0.98 (0.90-1.06)	1.51 (1.39-1.63)	0.71 (0.63-0.78)
GBS or Death	0.99 (0.91-1.07)	1.53 (1.41-1.66)	0.67 (0.60-0.75)
Insomnia or Death	0.95 (0.89-1.03)	1.45 (1.35-1.57)	0.73 (0.66-0.81)
Intracranial haemorrhage or Death	0.98 (0.91-1.07)	1.51 (1.39-1.64)	0.70 (0.63-0.78)
Ischaemic stroke or Death	0.97 (0.90-1.06)	1.51 (1.39-1.64)	0.71 (0.64-0.79)
Mood disorder or Death	0.99 (0.92-1.06)	1.32 (1.23-1.42)	0.82 (0.74-0.91)
Myoneural junction/muscle disease or Death	1.00 (0.92-1.08)	1.52 (1.40-1.64)	0.68 (0.62-0.76)
Nerve/Nerve root/Plexus disorder or Death	1.05 (0.98-1.12)	1.37 (1.28-1.47)	0.82 (0.75-0.90)
Parkinsonism or Death	0.97 (0.90-1.06)	1.53 (1.41-1.66)	0.68 (0.61-0.76)
Psychotic disorder or Death	0.97 (0.90-1.06)	1.53 (1.41-1.66)	0.69 (0.62-0.77)
Any first or Death	0.98 (0.92-1.05)	1.18 (1.10-1.26)	0.89 (0.80-0.98)
Anxiety disorder (any) or Death	1.01 (0.97-1.05)	1.13 (1.09-1.18)	0.98 (0.93-1.03)
Cognitive deficit (any) or Death	0.99 (0.94-1.04)	1.21 (1.15-1.28)	0.87 (0.81-0.94)
Epilepsy or seizures (any) or Death	0.97 (0.91-1.03)	1.35 (1.26-1.44)	0.82 (0.75-0.89)
Insomnia (any) or Death	0.96 (0.91-1.02)	1.32 (1.25-1.41)	0.84 (0.78-0.91)
Intracranial haemorrhage (any) or Death	0.97 (0.90-1.05)	1.48 (1.36-1.60)	0.72 (0.65-0.79)
Ischaemic stroke (any) or Death	0.98 (0.91-1.05)	1.40 (1.30-1.50)	0.75 (0.68-0.82)
Mood disorder (any) or Death	1.01 (0.97-1.05)	1.14 (1.10-1.20)	1.02 (0.96-1.08)
Psychotic disorder (any) or Death	0.98 (0.91-1.05)	1.44 (1.33-1.55)	0.75 (0.68-0.83)
Any or Death	1.00 (0.97-1.03)	1.09 (1.05-1.12)	1.03 (0.99-1.07)

Table S21 – Overlap in the follow-up time windows between the cohorts diagnosed just after vs. just before the emergence of a variant. The high overlap indicates that for each variant, both cohorts were after by similar contextual factors.

Variant	Follow-up overlap [%]
After vs. before alpha emergence	73.9
After vs. before delta emergence	65.3
After vs. before omicron emergence	82.9

Table S22 – Relative risk of any first neurological or psychiatric diagnosis being made just after vs. just before the emergence of a variant in the general population of the TriNetX US Collaborative Network. Values close to 1 indicate that little change has been observed in the frequency with which these diagnoses were made in the general population during the follow-up time windows corresponding to the different variants.

Variant	Incidence of diagnoses in the general population (RR)
After vs. before alpha emergence	0.98
After vs. before delta emergence	0.96
After vs. before omicron emergence	1.005

The RECORD statement – checklist of items, extended from the STROBE statement, that should be reported in observational studies using routinely collected health data.

	Item No.	STROBE items	Location in manuscript where items are reported	RECORD items	Location in manuscript where items are reported
**Title and abstract**					
	1	(a) Indicate the study's design with a commonly used term in the title or the abstract (b) Provide in the abstract an informative and balanced summary of what was done and what was found	Title and abstract	RECORD 1.1: The type of data used should be specified in the title or abstract. When possible, the name of the databases used should be included.<br><br>RECORD 1.2: If applicable, the geographic region and timeframe within which the study took place should be reported in the title or abstract.<br><br>RECORD 1.3: If linkage between databases was conducted for the study, this should be clearly stated in the title or abstract.	Title and abstract<br><br>Title and abstract<br><br>N/A
**Introduction**					
Background rationale	2	Explain the scientific background and rationale for the investigation being reported	Introduction		
Objectives	3	State specific objectives, including any prespecified hypotheses	Introduction		
**Methods**					
Study Design	4	Present key elements of study design early in the paper	Methods ('Study design and data collection')		
Setting	5	Describe the setting, locations, and relevant dates, including periods of recruitment, exposure, follow-up, and data collection	Methods ('Study design and data collection', 'Outcomes') as well as appendix		
Participants	6	*(a) Cohort study* - Give the eligibility criteria, and the sources and methods of selection of participants. Describe methods of follow-up<br>*Case-control study* - Give the eligibility criteria, and the sources and methods of case ascertainment and control selection. Give the rationale for the choice of cases and controls	Methods ('Cohorts') and appendix	RECORD 6.1: The methods of study population selection (such as codes or algorithms used to identify subjects) should be listed in detail. If this is not possible, an explanation should be provided.<br><br>RECORD 6.2: Any validation studies of the codes or algorithms used to select the population should be referenced. If validation was conducted for this study and not published	Methods ('Cohorts') and appendix<br><br>Methods ('Cohorts') and appendix

		Cross-sectional study - Give the eligibility criteria, and the sources and methods of selection of participants<br><br>*(b) Cohort study* - For matched studies, give matching criteria and number of exposed and unexposed<br>*Case-control study* - For matched studies, give matching criteria and the number of controls per case		elsewhere, detailed methods and results should be provided.<br><br>RECORD 6.3: If the study involved linkage of databases, consider use of a flow diagram or other graphical display to demonstrate the data linkage process, including the number of individuals with linked data at each stage.	N/A
Variables	7	Clearly define all outcomes, exposures, predictors, potential confounders, and effect modifiers. Give diagnostic criteria, if applicable.	Methods ('Outcomes') and appendix	RECORD 7.1: A complete list of codes and algorithms used to classify exposures, outcomes, confounders, and effect modifiers should be provided. If these cannot be reported, an explanation should be provided.	Methods ('Outcomes') and appendix
Data sources/ measurement	8	For each variable of interest, give sources of data and details of methods of assessment (measurement). Describe comparability of assessment methods if there is more than one group	Methods ('Cohorts', 'Outcomes', 'Covariates') and appendix		
Bias	9	Describe any efforts to address potential sources of bias	Methods ('Cohorts', 'Covariates', 'Statistical analysis') and appendix		
Study size	10	Explain how the study size was arrived at	Methods ('Cohorts')		
Quantitative variables	11	Explain how quantitative variables were handled in the analyses. If applicable, describe which groupings were chosen, and why	Methods ('Cohorts', 'Outcomes', 'Covariates')		
Statistical methods	12	(a) Describe all statistical methods, including those used to control for confounding<br>(b) Describe any methods used to examine subgroups and interactions<br>(c) Explain how missing data were addressed<br>(d) *Cohort study* - If applicable, explain how loss to follow-up was addressed<br>*Case-control study* - If applicable, explain how matching of cases and controls was addressed<br>*Cross-sectional study* - If applicable, describe analytical methods taking account of sampling strategy<br>(e) Describe any sensitivity analyses	Methods ('Statistical analysis') and appendix		

Data access and cleaning methods		..		RECORD 12.1: Authors should describe the extent to which the investigators had access to the database population used to create the study population.<br><br>RECORD 12.2: Authors should provide information on the data cleaning methods used in the study.	Author contributions<br><br>Appendix
Linkage		..		RECORD 12.3: State whether the study included person-level, institutional-level, or other data linkage across two or more databases. The methods of linkage and methods of linkage quality evaluation should be provided.	N/A
**Results**					
Participants	13	(a) Report the numbers of individuals at each stage of the study (*e.g.*, numbers potentially eligible, examined for eligibility, confirmed eligible, included in the study, completing follow-up, and analysed)<br>(b) Give reasons for non-participation at each stage.<br>(c) Consider use of a flow diagram	Results (first paragraph), Table 1	RECORD 13.1: Describe in detail the selection of the persons included in the study (*i.e.,* study population selection) including filtering based on data quality, data availability and linkage. The selection of included persons can be described in the text and/or by means of the study flow diagram.	Results (first paragraph), Table 1, Table S1-S4, S7-S9
Descriptive data	14	(a) Give characteristics of study participants (*e.g.*, demographic, clinical, social) and information on exposures and potential confounders<br>(b) Indicate the number of participants with missing data for each variable of interest<br>(c) *Cohort study* - summarise follow-up time (*e.g.*, average and total amount)	Table 1, Tables S1-S4, S7-S9		Table 1, Tables S1-S4, S7-S9
Outcome data	15	*Cohort study* - Report numbers of outcome events or summary measures over time<br>*Case-control study* - Report numbers in each exposure category, or summary measures of exposure<br>*Cross-sectional study* - Report numbers of outcome events or summary measures	Table 2, Fig. 1, Fig. S1-S4, Table S5		
Main results	16	(a) Give unadjusted estimates and, if applicable, confounder-adjusted estimates and their precision (e.g., 95%	Results and Table 2, Fig. 1-4, Figs. S1-S4, Table S5, Table S10		

		confidence interval). Make clear which confounders were adjusted for and why they were included<br>(b) Report category boundaries when continuous variables were categorized<br>(c) If relevant, consider translating estimates of relative risk into absolute risk for a meaningful time period			
Other analyses	17	Report other analyses done—e.g., analyses of subgroups and interactions, and sensitivity analyses	Results, Fig. 2-4, Fig. S2-S4, Table S10.		
**Discussion**					
Key results	18	Summarise key results with reference to study objectives	Discussion, first paragraph		
Limitations	19	Discuss limitations of the study, taking into account sources of potential bias or imprecision. Discuss both direction and magnitude of any potential bias	Discussion and appendix	RECORD 19.1: Discuss the implications of using data that were not created or collected to answer the specific research question(s). Include discussion of misclassification bias, unmeasured confounding, missing data, and changing eligibility over time, as they pertain to the study being reported.	Discussion and appendix
Interpretation	20	Give a cautious overall interpretation of results considering objectives, limitations, multiplicity of analyses, results from similar studies, and other relevant evidence	Discussion		
Generalisability	21	Discuss the generalisability (external validity) of the study results	Discussion		
**Other Information**					
Funding	22	Give the source of funding and the role of the funders for the present study and, if applicable, for the original study on which the present article is based	Abstract, Methods ("Role of the funding Source"), and Acknowledgements		
Accessibility of protocol, raw data, and programming code		..		RECORD 22.1: Authors should provide information on how to access any supplemental information such as the study protocol, raw data, or programming code.	Data sharing section

*Reference: Benchimol EI, Smeeth L, Guttmann A, Harron K, Moher D, Petersen I, Sørensen HT, von Elm E, Langan SM, the RECORD Working Committee. The REporting of studies Conducted using Observational Routinely-collected health Data (RECORD) Statement. *PLoS Medicine* 2015; **12** (10): e1001885. .

*Checklist is protected under Creative Commons Attribution (CC BY) license.
